# Nintedanib and immunomodulatory therapies in progressive fibrosing interstitial lung diseases

**DOI:** 10.1186/s12931-021-01668-1

**Published:** 2021-03-16

**Authors:** Vincent Cottin, Luca Richeldi, Ivan Rosas, Maria Otaola, Jin Woo Song, Sara Tomassetti, Marlies Wijsenbeek, Manuela Schmitz, Carl Coeck, Susanne Stowasser, Rozsa Schlenker-Herceg, Martin Kolb, the INBUILD Trial Investigators

**Affiliations:** 1 National Reference Center for Rare Pulmonary Diseases, Louis Pradel Hospital, Hospices Civils de Lyon, Claude Bernard University Lyon 1, University of Lyon, INRA, UMR754, Lyon, France; 2 Fondazione Policlinico A. Gemelli IRCCS, Università Cattolica del Sacro Cuore, Rome, Italy; 3 Baylor College of Medicine, Houston, TX USA; 4 Fundación Para El Estudio de Enfermedades Fibrosantes del Pulmón, Buenos Aires, Argentina; 5 Pulmonary and Critical Care Medicine, Asan Medical Center, University of Ulsan College of Medicine, Seoul, South Korea; 6 Department of Experimental and Clinical Medicine, Careggi University Hospital, Florence, Italy; 7 Department of Respiratory Medicine, Erasmus MC, University Medical Centre, Rotterdam, The Netherlands; 8 Mainanalytics GmbH, Sulzbach, Germany; 9 SCS Boehringer Ingelheim Comm.V., Brussels, Belgium; 10 Boehringer Ingelheim International GmbH, Ingelheim am Rhein, Germany; 11 Boehringer Ingelheim Pharmaceuticals, Inc., Ridgefield, CT USA; 12 McMaster University and St. Joseph’s Healthcare, Hamilton, ON Canada

**Keywords:** Pulmonary fibrosis, Connective tissue diseases, Autoimmune diseases, Corticosteroids, Immunosuppressants

## Abstract

**Background:**

In the INBUILD trial in patients with chronic fibrosing interstitial lung diseases (ILDs) and a progressive phenotype, nintedanib reduced the rate of ILD progression with adverse events that were manageable for most patients. We investigated the potential impact of immunomodulatory therapies on the efficacy and safety of nintedanib.

**Methods:**

Subjects with fibrosing ILDs other than idiopathic pulmonary fibrosis, who had shown progression of ILD within the prior 24 months despite management in clinical practice, were randomized to receive nintedanib or placebo. Certain immunomodulatory therapies were restricted for the first 6 months. We analyzed post-hoc the rate of decline in forced vital capacity (FVC) over 52 weeks in subgroups by glucocorticoid use at baseline and in analyses excluding subjects or FVC measurements taken after initiation of restricted immunomodulatory or antifibrotic therapies.

**Results:**

Of 663 subjects, 361 (54.4%) were taking glucocorticoids at baseline (353 at a dose of ≤ 20 mg/day). In the placebo group, the adjusted rate of decline in FVC (mL/year) over 52 weeks was numerically greater in subjects taking than not taking glucocorticoids at baseline (− 206.4 [SE 20.2] vs − 165.8 [21.9]). The difference between the nintedanib and placebo groups was 133.3 (95% CI 76.6, 190.0) mL/year in subjects taking glucocorticoids at baseline and 76.1 (15.0, 137.2) mL/year in subjects who were not (interaction *P* = 0.18). The effect of nintedanib on reducing the rate of FVC decline in analyses excluding subjects or measurements taken after initiation of restricted immunomodulatory or antifibrotic therapies was similar to the primary analysis. The adverse event profile of nintedanib was similar between subjects who did and did not use prohibited or restricted therapies at baseline or during treatment with trial drug.

**Conclusions:**

In patients with progressive fibrosing ILDs, the effect of nintedanib on reducing FVC decline was not influenced by the use of immunomodulatory therapies. Nintedanib can be used in combination with immunomodulatory therapies in patients with progressive fibrosing ILDs.

*Trial registration* ClinicalTrials.gov, NCT02999178. Registered 21 December 2016, https://clinicaltrials.gov/ct2/show/NCT02999178

**Supplementary Information:**

The online version contains supplementary material available at 10.1186/s12931-021-01668-1.

## Introduction

Idiopathic pulmonary fibrosis (IPF) is, by definition, a progressive fibrosing interstitial lung disease (ILD) [1]. In addition to IPF, a proportion of patients with other chronic fibrosing ILDs develop a progressive phenotype characterized by increasing fibrosis on high resolution computed tomography (HRCT), worsening of lung function, symptoms and quality of life, and early mortality [2–5].

Immunosuppressants are the mainstay of treatment for autoimmune diseases such as rheumatoid arthritis (RA) and systemic sclerosis (SSc). Immunomodulatory medications such as glucocorticoids are also frequently used in the treatment of other non-IPF ILDs such as hypersensitivity pneumonitis, idiopathic non-specific interstitial pneumonia (iNSIP) and unclassifiable idiopathic interstitial pneumonia (IIP) [6–8].

In the INBUILD trial in subjects with non-IPF chronic fibrosing ILDs that were progressive despite management deemed appropriate in clinical practice, nintedanib slowed the progression of fibrosing ILD, as measured by the annual rate of decline in forced vital capacity (FVC), both in the overall population and in the co-primary population of subjects with a usual interstitial pneumonia (UIP)-like fibrotic pattern on HRCT [9]. Although the INBUILD trial was not powered to study individual ILDs, subgroup analyses suggested that nintedanib had a consistent effect on FVC decline across diagnostic groups [10].

We investigated the therapies that are often used to treat non-IPF ILDs in clinical practice that were used at baseline and during the INBUILD trial, and the potential impact of these therapies on the treatment effect of nintedanib.

## Methods

### Trial design

The INBUILD trial design has been described and the protocol is publicly available [9]. Briefly, subjects had a physician-diagnosed chronic fibrosing ILD other than IPF, reticular abnormality with traction bronchiectasis (with or without honeycombing) of > 10% extent on HRCT, FVC ≥ 45% predicted and diffusion capacity of the lung for carbon monoxide (DLco) ≥ 30–< 80% predicted. Subjects met one of the following criteria for ILD progression within the 24 months before screening, despite management deemed appropriate in clinical practice: relative decline in FVC ≥ 10% predicted; relative decline in FVC ≥ 5–< 10% predicted and worsened respiratory symptoms; relative decline in FVC ≥ 5–< 10% predicted and increased extent of fibrosis on HRCT; worsened respiratory symptoms and increased extent of fibrosis on HRCT [9]. Subjects were randomized to receive nintedanib 150 mg bid or placebo, stratified by fibrotic pattern on HRCT (UIP-like fibrotic pattern or other fibrotic patterns) [9]. The protocol was approved by an independent ethics committee or institutional review board at each participating center (additional details can be found in Additional file 1: Appendix S1).

### Restricted and prohibited immunomodulatory and antifibrotic therapies

Restricted therapies were azathioprine, cyclosporine, mycophenolate mofetil, tacrolimus, oral glucocorticoids > 20 mg/day, or the combination of oral glucocorticoids, azathioprine and *N*-acetylcysteine (not permitted within 4 weeks of randomization); cyclophosphamide (not permitted within 8 weeks of randomization); and rituximab (not permitted within 6 months of randomization). Investigators were asked not to consider patients with autoimmune disease that was managed using any of these restricted therapies for participation in the trial. Patients who took a restricted therapy to treat their ILD, and whose ILD was progressing, could participate in the trial if the restricted therapy was discontinued. Apart from the restricted therapies listed above, there was no limit on the use of stable doses of biologic or non-biologic disease-modifying anti-rheumatic drugs (DMARDs). Initiation of the restricted therapies was allowed after 6 months of trial treatment in subjects with deterioration of ILD or CTD. Use of nintedanib and pirfenidone was prohibited at randomization and during the trial.

Restricted or prohibited therapies were defined based on customized drug groupings (CDGs). We present data based on the CDGs “corticosteroids”, “nintedanib” and “pirfenidone”, and the preferred names azathioprine, cyclosporine, mycophenolate mofetil, tacrolimus, glucocorticoids, cyclophosphamide, and rituximab. Glucocorticoids were only counted as restricted therapies if a high dose (> 20 mg/day prednisone or equivalent) was used and the route of administration was oral, intravenous, intravenous bolus, intravenous drip, or intramuscular. DMARDs were defined based on WHO standardized drug groupings (SDGs).

### Analyses

Analyses of the annual rate of decline in FVC were consistent with previous analyses [9, 10] and are summarized in Additional file 2: Appendix S2. We analyzed the rate of decline in FVC (mL/year) over 52 weeks in subgroups taking glucocorticoids: > 20 mg/day (referred to as “high-dose”) (in deviation from the protocol) or ≤ 20 mg/day prednisone or equivalent (referred to as “low-dose”) or not taking glucocorticoids at baseline. We analyzed potential heterogeneity in the relative effect of nintedanib in reducing the rate of decline in FVC between these subgroups (see Additional file 2). We also analyzed the rate of decline in FVC in subgroups by use of high-dose glucocorticoids, low-dose glucocorticoids, or no glucocorticoids at baseline.

To assess the potential impact of prohibited or restricted therapies, including high-dose glucocorticoids, on the treatment effect of nintedanib, we analyzed the rate of decline in FVC over 52 weeks (1) excluding subjects who took ≥ 1 prohibited or restricted therapy over 52 weeks and (2) excluding FVC measurements taken after initiation of prohibited or restricted therapy. Use of prohibited or restricted therapies over 52 weeks comprised use at baseline, during treatment with trial drug, or following discontinuation of trial drug (up to week 52) for any duration. We report adverse events in subgroups who did and did not use prohibited or restricted therapies at baseline or during treatment with trial drug. Finally, we analyzed the rate of decline in FVC over 52 weeks (1) excluding subjects who took ≥ 1 low-dose glucocorticoid or prohibited or restricted therapy over 52 weeks and (2) excluding FVC measurements after initiation of these therapies. Analyses were post-hoc and performed in all subjects, subjects with a UIP-like fibrotic pattern on HRCT, and subjects with other fibrotic patterns on HRCT with the exception of the last analyses and adverse events, which were only investigated in the overall population. Analyses were performed using SAS version 9.4.

## Results

### Subjects

A total of 663 subjects received ≥ 1 dose of trial drug. At baseline, mean (SD) age was 65.8 (9.8) years, FVC was 69.0 (15.6) % predicted; 53.7% of subjects were male and 62.1% had a UIP-like fibrotic pattern on HRCT. Subjects’ ILD diagnoses (grouped) were chronic hypersensitivity pneumonitis (26.1%), autoimmune disease-related ILDs (25.6%) (13.4% had RA-ILD), iNSIP (18.9%), unclassifiable IIP (17.2%) and other ILDs (12.2%).

At baseline, 353 subjects (53.2%) (174 nintedanib, 179 placebo) were taking low-dose glucocorticoids, while 8 subjects (3 nintedanib, 5 placebo) were taking high-dose glucocorticoids; 4.7% of subjects were taking biologic DMARDs and 11.6% of subjects were taking non-biologic DMARDs (Additional file 3: Table S1). Most subjects taking DMARDs had autoimmune disease-related ILDs (Additional file 4: Table S2).

### Analyses based on glucocorticoid use at baseline

Baseline characteristics of subjects taking high-dose, low-dose, or no glucocorticoids are shown in Table 1 (overall population) and Additional file 5: Table S3 and Additional file 6: Table S4 (subpopulations by HRCT pattern).

**Table 1:** Baseline characteristics of subgroups taking high-dose, low-dose, or no glucocorticoids at baseline

	High-dose glucocorticoids (n = 8)	Low-dose glucocorticoids (n = 353)	No glucocorticoids (n = 302)
Male	2 (25.0)	189 (53.5)	165 (54.6)
Age, years	61.8 (4.2)	65.5 (10.0)	66.2 (9.6)
Former or current smoker	3 (37.5)	173 (49.0)	162 (53.6)
ILD diagnosis			
Hypersensitivity pneumonitis	5 (62.5)	96 (27.2)	72 (23.8)
Autoimmune ILDs^a^	1 (12.5)	114 (32.3)	55 (18.2)
iNSIP	0	65 (18.4)	60 (19.9)
Unclassifiable IIP	2 (25.0)	41 (11.6)	71 (23.5)
Other ILDs^b^	0	37 (10.5)	44 (14.6)
FVC, mL	1744 (546)	2287 (696)	2397 (771)
FVC, % predicted	53.8 (6.9)	67.4 (14.9)	71.2 (16.2)
DLco, % predicted	44.4 (9.1)	44.4 (12.6)	48.2 (14.6)

Data are shown as n (%) or mean (SD). Glucocorticoids with oral, intravenous, intravenous bolus, intravenous drip, or intramuscular route of administration. High-dose glucocorticoids: > 20 mg/day prednisone or equivalent

DLco: diffusing capacity of the lung for carbon monoxide, corrected for hemoglobin; FVC: forced vital capacity; IIP: idiopathic interstitial pneumonia; ILD: interstitial lung disease; iNSIP: idiopathic non-specific interstitial pneumonia; MCTD: mixed connective tissue disease; RA: rheumatoid arthritis; SSc: systemic sclerosis

^a^Included RA-ILD, SSc-ILD, MCTD-ILD, plus autoimmune ILDs in the “Other fibrosing ILDs” category of the case report form

^b^Included sarcoidosis, exposure-related ILDs and selected other terms in the “Other fibrosing ILDs” category of the case report form

**Fig. 1:**
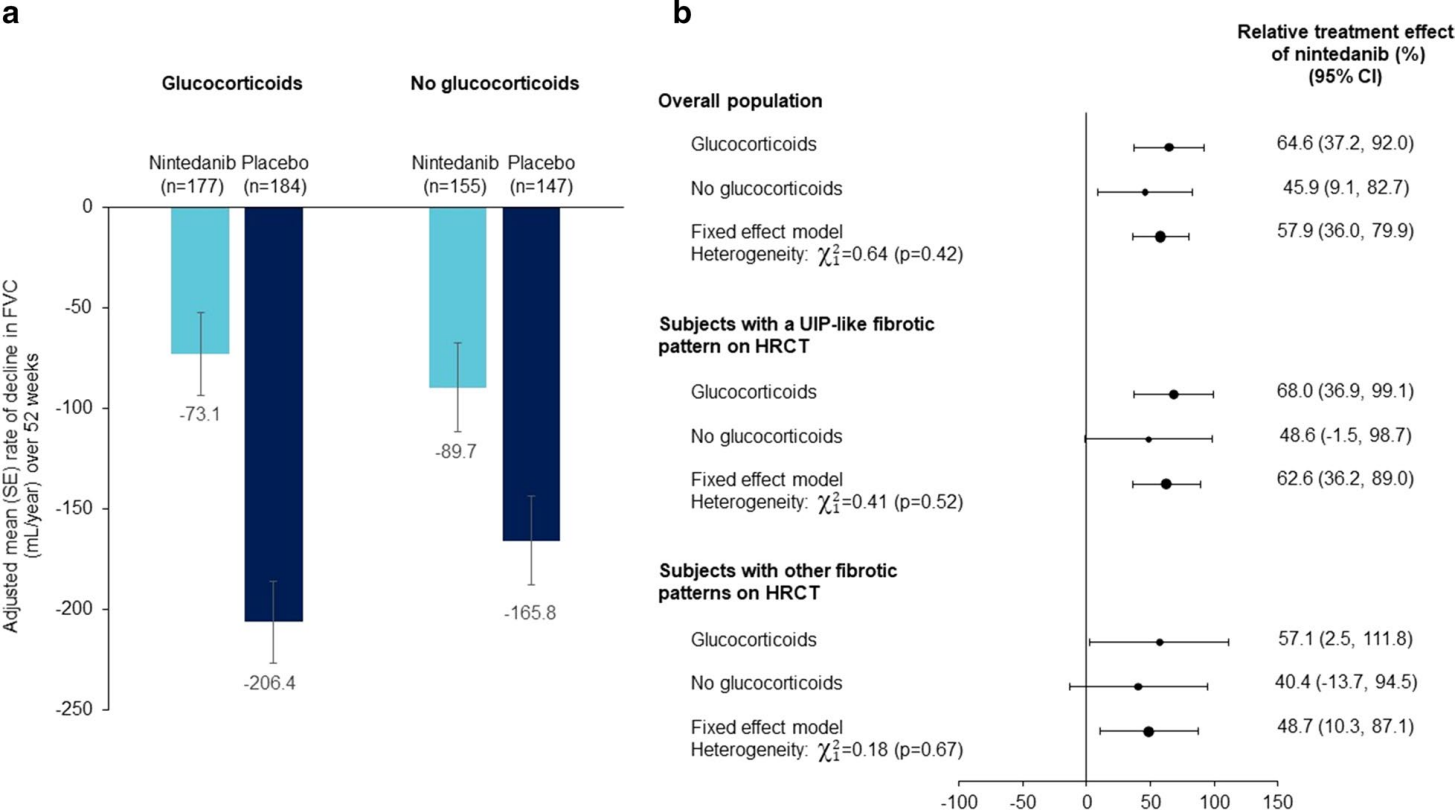
Rate of decline in forced vital capacity (FVC) (mL/year) with nintedanib and placebo (**a**) and relative treatment effect of nintedanib (**b**) over 52 weeks in subgroups taking or not taking glucocorticoids at baseline. Glucocorticoids were taken at a dose of > 20 mg/day prednisone or equivalent by 8 subjects. *HRCT* high-resolution computed tomography, *UIP* usual interstitial pneumonia

In the overall population, the adjusted rate of decline in FVC (mL/year) over 52 weeks in the placebo group was numerically greater in subjects taking glucocorticoids (high-dose [n = 8] or low-dose [n = 353]) at baseline than in those not taking glucocorticoids (− 206.4 [SE 20.2] vs − 165.8 [21.9]) (Fig. 1a). Findings were similar in subjects with a UIP-like fibrotic pattern on HRCT (− 254.1 [28.4] vs − 164.6 [29.5] mL/year) (Additional file 7: Table S5). In subjects with other fibrotic patterns on HRCT, the adjusted rate of decline in FVC (mL/year) over 52 weeks was − 145.0 (27.9) in those taking glucocorticoids and − 167.0 (32.8) in those not taking glucocorticoids at baseline (Additional file 7: Table S5). The interaction* P* value did not indicate heterogeneity in the treatment effect of nintedanib on reducing the rate of decline in FVC between the subgroups by use versus non-use of glucocorticoids (high-dose or low-dose) at baseline in the overall population (*P* = 0.18), in subjects with a UIP-like fibrotic pattern on HRCT (*P* = 0.11) or in subjects with other fibrotic patterns on HRCT (*P* = 0.80) (Additional file 8: Figure S1). There was also no indication of heterogeneity in the relative treatment effect of nintedanib between subgroups by use of glucocorticoids (high-dose or low-dose) in the overall population or in the subpopulations by HRCT pattern (Fig. 1b).

In analyses in subgroups by use of high-dose glucocorticoids, low-dose glucocorticoids, or no glucocorticoids at baseline, the interaction* P* values did not indicate heterogeneity in the treatment effect of nintedanib on reducing the rate of decline in FVC between the subgroups in the overall population or in the subpopulations by HRCT pattern (Additional file 9: Figure S2).

### Use of prohibited or restricted therapies over 52 weeks

The proportions of subjects taking prohibited or restricted therapies over 52 weeks were smaller in the nintedanib group than the placebo group in the overall population (16.0% vs 27.5%), in subjects with a UIP-like fibrotic pattern on HRCT (17.0% vs 28.2%), in subjects with other fibrotic patterns on HRCT (14.3% vs 26.4%) and across subgroups by ILD diagnosis (Table 2; Additional file 10: Table S6, Additional file 11: Table S7, Additional file 12: Table S8). The most frequently used restricted therapies were high-dose glucocorticoids (13.3% nintedanib, 21.8% placebo) and mycophenolate mofetil (2.7% in both groups) (Table 2). Over 52 weeks, in the overall population, low-dose glucocorticoids were taken by a higher proportion of subjects in the nintedanib group than the placebo group (52.4% vs 45.9%). Findings were similar between the subpopulations by HRCT pattern (Additional file 13: Table S9).

**Table 2:** Restricted or prohibited immunomodulatory or antifibrotic therapies taken at baseline, during treatment with trial drug and/or following discontinuation of trial drug over 52 weeks by customized drug grouping or preferred name

	Nintedanib (n = 332)	Placebo (n = 331)
≥ 1 restricted or prohibited therapy	53 (16.0)	91 (27.5)
Glucocorticoids^a^	44 (13.3)	72 (21.8)
Mycophenolate mofetil	9 (2.7)	9 (2.7)
Azathioprine	4 (1.2)	6 (1.8)
Tacrolimus	4 (1.2)	5 (1.5)
Ciclosporin	1 (0.3)	6 (1.8)
Rituximab	3 (0.9)	2 (0.6)
Cyclophosphamide	0 (0.0)	3 (0.9)
Nintedanib^a^	0 (0.0)	3 (0.9)
Pirfenidone^a^	2 (0.6)	1 (0.3)

Data are n (%) of subjects who took ≥ 1 such therapy at baseline, during treatment with trial drug, and/or following discontinuation of trial drug (up to week 52) for any duration. Glucocorticoids were only counted as restricted therapies if used at high dose (> 20 mg/day prednisone or equivalent) and if the route of administration was oral, intravenous, intravenous bolus, intravenous drip, or intramuscular. Other therapies are displayed regardless of dose or route of administration

^a^Based on customized drug grouping; for other therapies, preferred names are shown

**Fig. 2:**
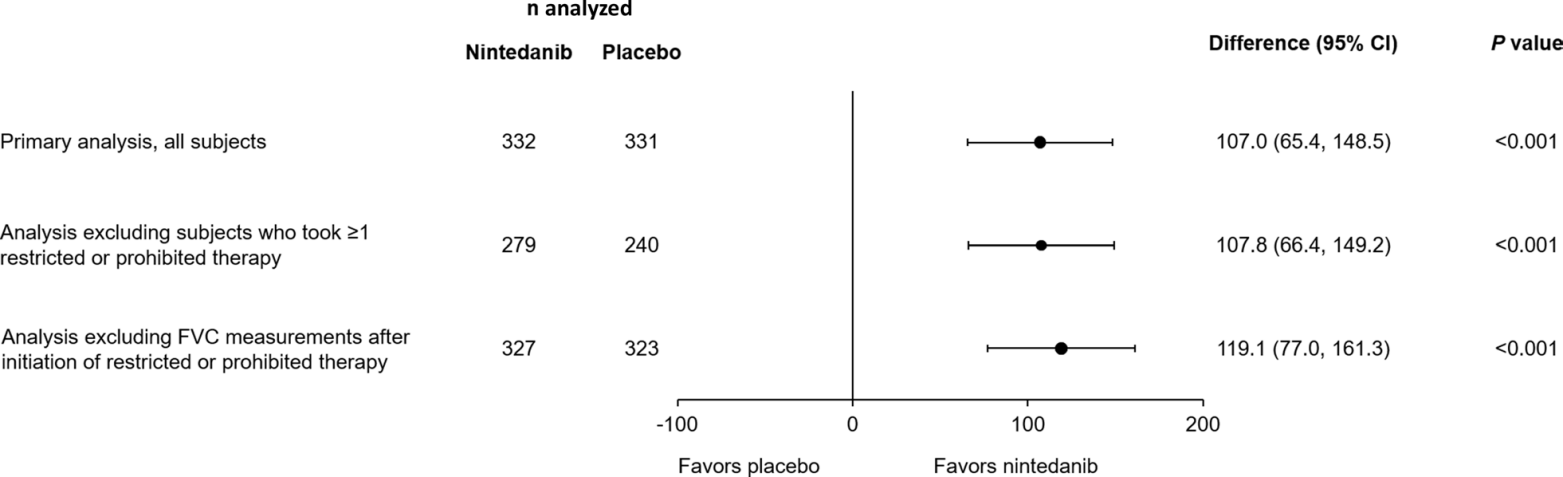
Rate of decline in forced vital capacity (FVC) (mL/year) over 52 weeks in the primary analysis, in an analysis excluding subjects who took ≥ 1 restricted or prohibited therapy, and in an analysis excluding FVC measurements taken after initiation of restricted or prohibited therapy (in the overall population)

In the overall population, the effect of nintedanib versus placebo in reducing the rate of decline in FVC in analyses excluding subjects who took ≥ 1 prohibited or restricted therapy over 52 weeks, or excluding FVC measurements taken after initiation of prohibited or restricted therapy, were similar to the primary analysis (Fig. 2). These findings were also observed in the subpopulations by HRCT pattern (Additional file 14: Figure S3).

**Fig. 3:**
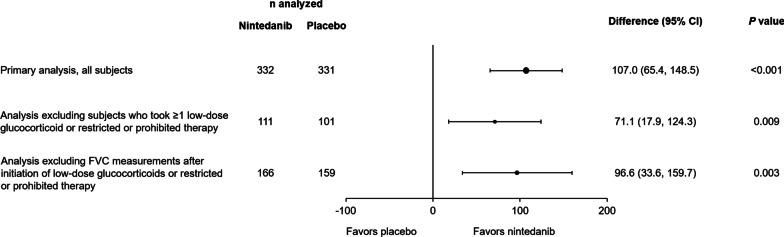
Rate of decline in forced vital capacity (FVC) (mL/year) over 52 weeks in the primary analysis, in an analysis excluding subjects who took ≥ 1 low-dose glucocorticoid or restricted or prohibited therapy, and in an analysis excluding FVC measurements taken after initiation of low-dose glucocorticoids or restricted or prohibited therapy (in the overall population)

Similarly, in the overall population, the effect of nintedanib versus placebo in reducing the rate of decline in FVC was maintained in an analysis excluding subjects who took ≥ 1 low-dose glucocorticoid or prohibited or restricted therapy over 52 weeks, or excluding FVC measurements taken after initiation of these therapies (Fig. 3).

### Adverse events by use of prohibited or restricted therapies at baseline or during treatment with trial drug

The adverse event profile of nintedanib was similar between subjects who did and did not use prohibited or restricted therapies at baseline or during treatment with trial drug (Table 3). Lower respiratory tract infections (e.g. bronchitis), adverse events of ILD progression and dyspnea, and serious adverse events were more frequently reported in both treatment groups in subjects who used than did not use prohibited or restricted medications over 52 weeks (Table 3).

**Table 3:** Adverse events in subgroups by use of restricted or prohibited immunomodulatory or antifibrotic therapies

	Restricted/prohibited medication use		No restricted/prohibited medication use	
	Nintedanib (n = 39)	Placebo (n = 80)	Nintedanib (n = 293)	Placebo (n = 251)
Any adverse event	39 (100.0)	78 (97.5)	278 (94.9)	218 (86.9)
Most frequent adverse events^a^				
Diarrhea	27 (69.2)	19 (23.8)	195 (66.6)	60 (23.9)
Nausea	12 (30.8)	10 (12.5)	84 (28.7)	21 (8.4)
Bronchitis	14 (35.9)	20 (25.0)	27 (9.2)	27 (10.8)
Nasopharyngitis	3 (7.7)	10 (12.5)	41 (14.0)	30 (12.0)
Dyspnea	8 (20.5)	19 (23.8)	28 (9.6)	25 (10.0)
Vomiting	8 (20.5)	3 (3.8)	53 (18.1)	14 (5.6)
Cough	5 (12.8)	16 (20.0)	28 (9.6)	28 (11.2)
Decreased appetite	6 (15.4)	6 (7.5)	42 (14.3)	11 (4.4)
Headache	4 (10.3)	9 (11.3)	31 (10.6)	14 (5.6)
Alanine aminotransferase increased	6 (15.4)	5 (6.3)	37 (12.6)	7 (2.8)
Progression of ILD^b^	8 (20.5)	25 (31.3)	8 (2.7)	14 (5.6)
Weight decreased	4 (10.3)	3 (3.8)	37 (12.6)	8 (3.2)
Aspartate aminotransferase increased	6 (15.4)	5 (6.3)	32 (10.9)	7 (2.8)
Abdominal pain	3 (7.7)	2 (2.5)	31 (10.6)	6 (2.4)
Serious adverse event^c^	27 (69.2)	45 (56.3)	80 (27.3)	65 (25.9)
Fatal adverse event	3 (7.7)	8 (10.0)	8 (2.7)	9 (3.6)
Adverse event leading to permanent treatment discontinuation	8 (20.5)	10 (12.5)	57 (19.5)	24 (9.6)

Data are n (%) of patients with ≥ 1 such adverse event reported over 52 weeks (or until 28 days after last trial drug intake in patients who discontinued trial drug before week 52)

^a^Adverse events, coded using preferred terms in the Medical Dictionary for Regulatory Activities, reported in > 10% of patients in either treatment group in the overall population

^b^Based on the preferred term “interstitial lung disease” in the Medical Dictionary for Regulatory Activities

^c^Adverse event that resulted in death, was life-threatening, resulted in hospitalization or prolongation of hospitalization, resulted in persistent or clinically significant disability or incapacity, was a congenital anomaly or birth defect, or was deemed to be serious for any other reason

## Discussion

Subjects enrolled in the INBUILD trial had chronic fibrosing ILDs that had progressed within the previous 2 years despite management deemed appropriate in clinical practice. At the time the trial was conducted, there were no approved treatments for progressive fibrosing ILDs other than IPF, but glucocorticoids and immunomodulatory therapies were the mainstay of treatment for fibrosing ILDs [8] and at baseline, glucocorticoids were taken by over half the patients in the INBUILD trial. In the SENSCIS trial in patients with SSc-ILD, subjects were allowed to continue taking certain immunomodulatory medications. Data from this trial suggest that combination therapy with immunomodulatory therapy and nintedanib may provide the greatest benefit in reducing FVC decline in patients with SSc-ILD [11]. In the INBUILD trial, to minimize the potential impact of immunomodulatory therapies on the assessment of the efficacy and safety of nintedanib, the use of such therapies was restricted at randomization and over the first 6 months of the trial. The use of glucocorticoids at a dose of ≤ 20 mg/day prednisone or equivalent was permitted and over half the subjects were taking these at baseline. Nintedanib had a consistent effect on reducing the rate of decline in FVC in subjects who were and were not taking glucocorticoids at baseline, with a safety profile consistent with that observed in patients with IPF [12, 13].

In the placebo group, the rate of decline in FVC was numerically greater in subjects who were taking glucocorticoids at baseline than in those who were not. These data should be interpreted with caution given that there were differences between these subgroups at baseline; however, other studies have found no benefit of glucocorticoids in reducing the progression of fibrosing ILDs [6, 14–17]. The INBUILD trial was not designed to determine whether immunomodulatory therapies had adverse effects in patients with fibrosing ILDs, as observed in patients with IPF treated with prednisone and azathioprine in the PANTHER-IPF trial [15]. However, in both treatment groups, a higher proportion of subjects taking than not taking restricted therapies at baseline experienced lower respiratory infections such as bronchitis. This may have been due partly to an increased risk of infections in patients taking immunomodulatory medications, and partly to greater use of such medications in patients with more severe/progressive disease, as suggested by the lower FVC and DLco at baseline, greater rate of decline in FVC during the trial, and higher frequency of adverse events of dyspnea, ILD progression and serious adverse events in the subgroup taking glucocorticoids at baseline.

The proportion of subjects who took restricted immunomodulatory therapies during the INBUILD trial was higher in the placebo group than the nintedanib group, across subpopulations by HRCT pattern and subgroups by ILD diagnosis. As restricted medications were allowed as “rescue” therapy, i.e. in case of clinically significant deterioration, the increased use of such medications in the placebo group might be expected given the higher rate of FVC decline in this group. The potential impact of prohibited or restricted therapies on the treatment effect of nintedanib could not be determined by conducting a subgroup analysis as the use of such therapies is a post-baseline factor. Instead, we addressed this question firstly by excluding subjects who used these medications from the analysis, and secondly by excluding FVC measurements taken after these medications were initiated. Both of these methodologies were conservative, as they took into account use of any of these medications at any time, for any duration, and for any reason, and indicated that use of prohibited and restricted medications had no impact on the effect of nintedanib in reducing the rate of decline in FVC.

## Conclusions

The patients enrolled in the INBUILD trial had chronic fibrosing ILDs that were progressing despite management deemed appropriate in clinical practice, including immunomodulatory therapies commonly used in the treatment of ILDs. While the INBUILD trial was not designed to evaluate the effects of concomitant medications on the rate of decline in FVC, our analyses suggest that the use of glucocorticoids at baseline, or the introduction of immunomodulatory therapies during the trial, did not affect the benefit of nintedanib in reducing the rate of FVC decline in patients with chronic fibrosing ILDs and a progressive phenotype.

## Supplementary Information

**Additional file 1: Appendix S1.** List of independent ethics committees and institutional review boards.

**Additional file 2: Appendix S2.** Analysis of the annual rate of decline in FVC (mL/year).

**Additional file 3: Table S1.** Proportions of subjects taking disease-modifying anti-rheumatic drugs (DMARDs) at baseline by WHO standardized drug grouping and preferred name.

**Additional file 4: Table S2.** Proportions of subjects taking disease-modifying anti-rheumatic drugs (DMARDs) at baseline by WHO standardized drug grouping and preferred name in subgroups by ILD diagnosis.

**Additional file 5: Table S3.** Baseline characteristics of subjects with a UIP-like fibrotic pattern on HRCT taking high-dose, low-dose, or no glucocorticoids at baseline.

**Additional file 6: Table S4.** Baseline characteristics of subjects with other fibrotic patterns on HRCT taking high-dose, low-dose, or no glucocorticoids at baseline.

**Additional file 7: Table S5.** Rate of decline in forced vital capacity (FVC) (mL/year) over 52 weeks in subgroups taking or not taking glucocorticoids (high-dose or low-dose) at baseline in subjects with a UIP-like fibrotic pattern on HRCT and subjects with other fibrotic patterns on HRCT.

**Additional file 8: Figure S1.** Rate of decline in forced vital capacity (FVC) (mL/year) over 52 weeks in subgroups taking or not taking glucocorticoids (high-dose or low-dose) at baseline in the overall population (A), in subjects with a UIP-like fibrotic pattern on HRCT (B) and in subjects with other fibrotic patterns on HRCT (C).

**Additional file 9: Figure S2.** Rate of decline in forced vital capacity (FVC) (mL/year) over 52 weeks in subgroups taking high-dose, low-dose, or no glucocorticoids at baseline in the overall population (A), in subjects with a UIP-like fibrotic pattern on HRCT (B) and in subjects with other fibrotic patterns on HRCT (C).

**Additional file 10: Table S6.** Restricted or prohibited immunomodulatory or antifibrotic therapies taken at baseline, during treatment with trial drug and/or following discontinuation of trial drug over 52 weeks by customized drug grouping or preferred name in subjects with a UIP-like fibrotic pattern on HRCT.

**Additional file 11: Table S7.** Restricted or prohibited immunomodulatory or antifibrotic therapies taken at baseline, during treatment with trial drug and/or following discontinuation of trial drug over 52 weeks by customized drug grouping (CDG) category or preferred name in subjects with other fibrotic patterns on HRCT.

**Additional file 12: Table S8.** Restricted or prohibited immunomodulatory or antifibrotic therapies taken at baseline, during treatment with trial drug and/or following discontinuation of trial drug over 52 weeks by customized drug grouping or preferred name in subgroups by ILD diagnosis.

**Additional file 13: Table S9.** Proportions of subjects taking high-dose or low-dose glucocorticoids at baseline, during treatment and/or following discontinuation of trial drug over 52 weeks.

**Additional file 14: Figure S3.** Rate of decline in forced vital capacity (FVC) (mL/year) over 52 weeks in subjects with a UIP-like fibrotic pattern on HRCT (A) or other fibrotic patterns on HRCT (B) in the primary analysis, in an analysis excluding subjects who took ≥ 1 restricted or prohibited therapy, and in an analysis excluding FVC measurements taken after initiation of restricted or prohibited therapy.

## Data Availability

Researchers may request access to de-identified participant clinical study data with documentation describing the structure and content of the datasets. Upon approval, and governed by a Data Sharing Agreement, data would be shared in a secured data-access system for a period of 1 year, which may be extended upon request. Researchers should use the link https://trials.boehringer-ingelheim.com/ to request access to study data.

## Abbreviations

CDG: Customized drug grouping
DLco: Diffusing capacity of the lung for carbon monoxide
DMARD: Disease-modifying anti-rheumatic drug
FVC: Forced vital capacity
HRCT: High-resolution computed tomography
IIP: Idiopathic interstitial pneumonia
ILD: Interstitial lung disease
iNSIP: Idiopathic non-specific interstitial pneumonia
IPF: Idiopathic pulmonary fibrosis
RA: Rheumatoid arthritis
SDG: Standardized drug grouping
SSc: Systemic sclerosis
UIP: Usual interstitial pneumonia
