# Supplementary material for: Nintedanib and immunomodulatory therapies in progressive fibrosing interstitial lung diseases
Source: Respir Res. 2021 Mar 16;22:84. doi: 10.1186/s12931-021-01668-1 (PMC7962343; doi:10.1186/s12931-021-01668-1)
Supplement: Supplementary file 1 — Additional file 1: Appendix S1. List of independent ethics committees and institutional review boards. [file 12931_2021_1668_MOESM1_ESM.docx]

**Supplemental Appendix 1:** List of independent ethics committees and institutional review boards

The protocol was approved by an independent ethics committee or institutional review board at each participating center. The names of the independent ethics committees and institutional review boards that approved the protocol are listed here.

**
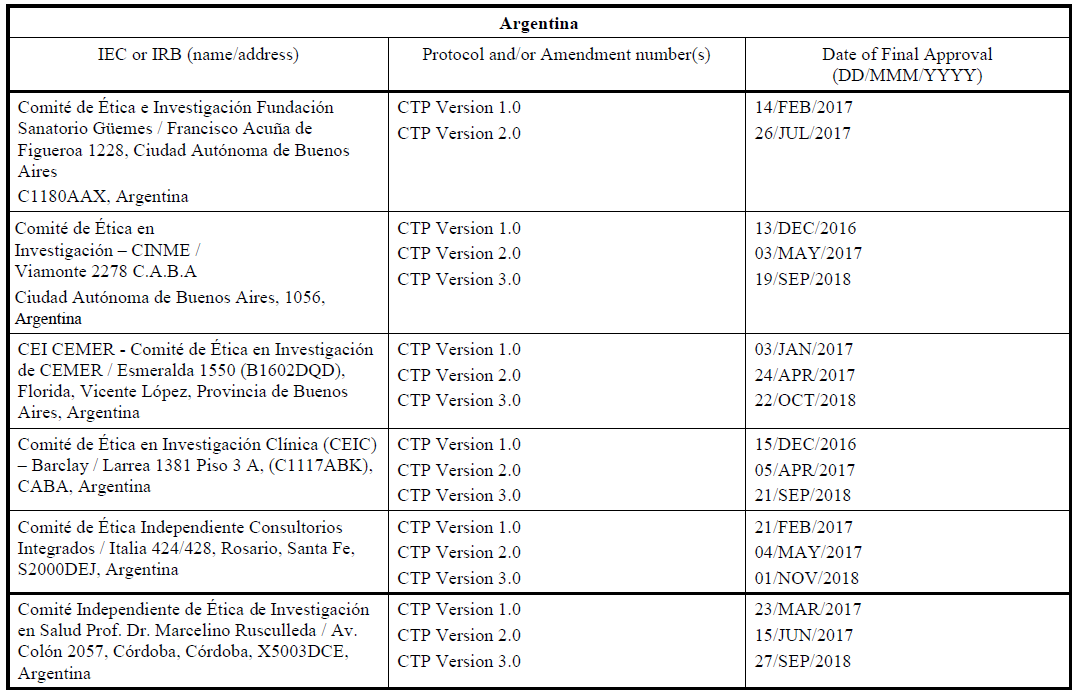
**

**
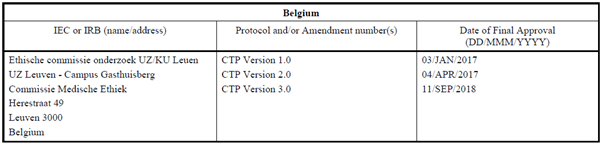
**


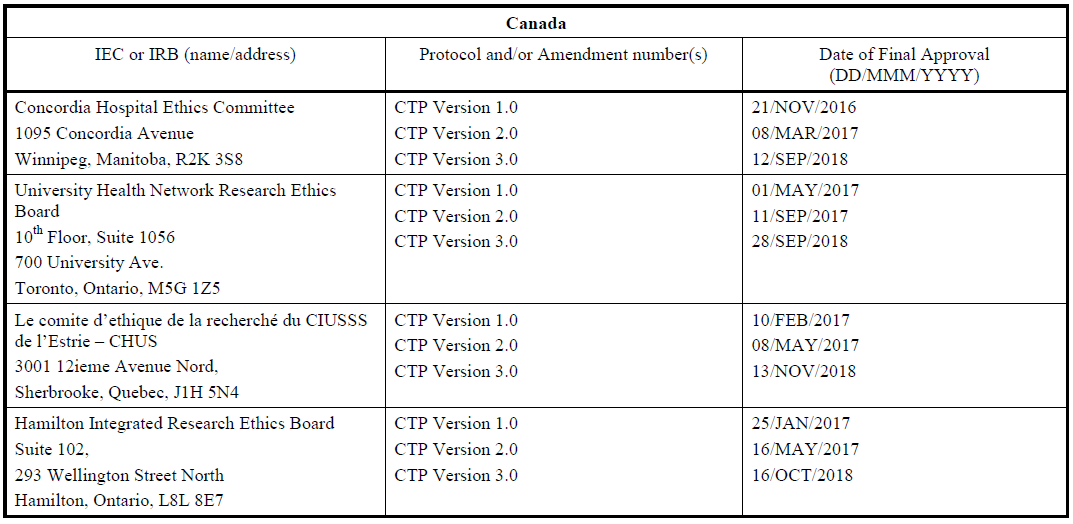


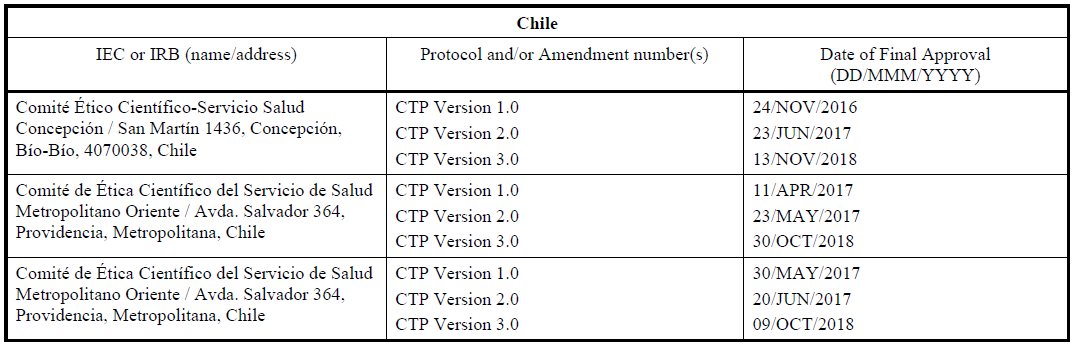


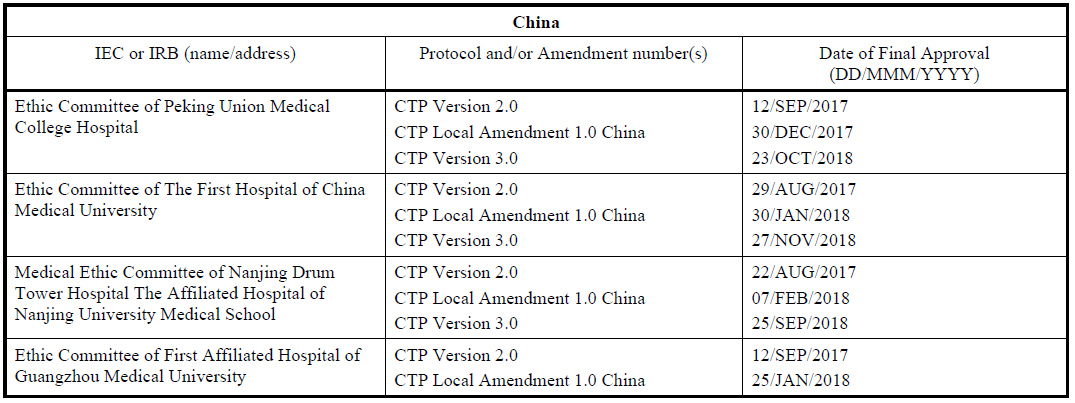


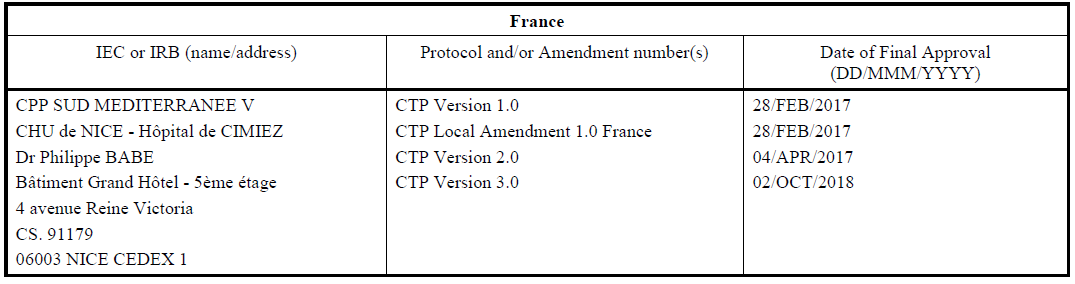


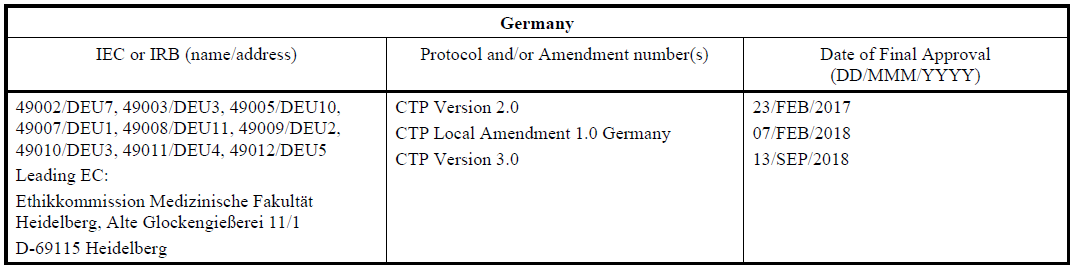


**
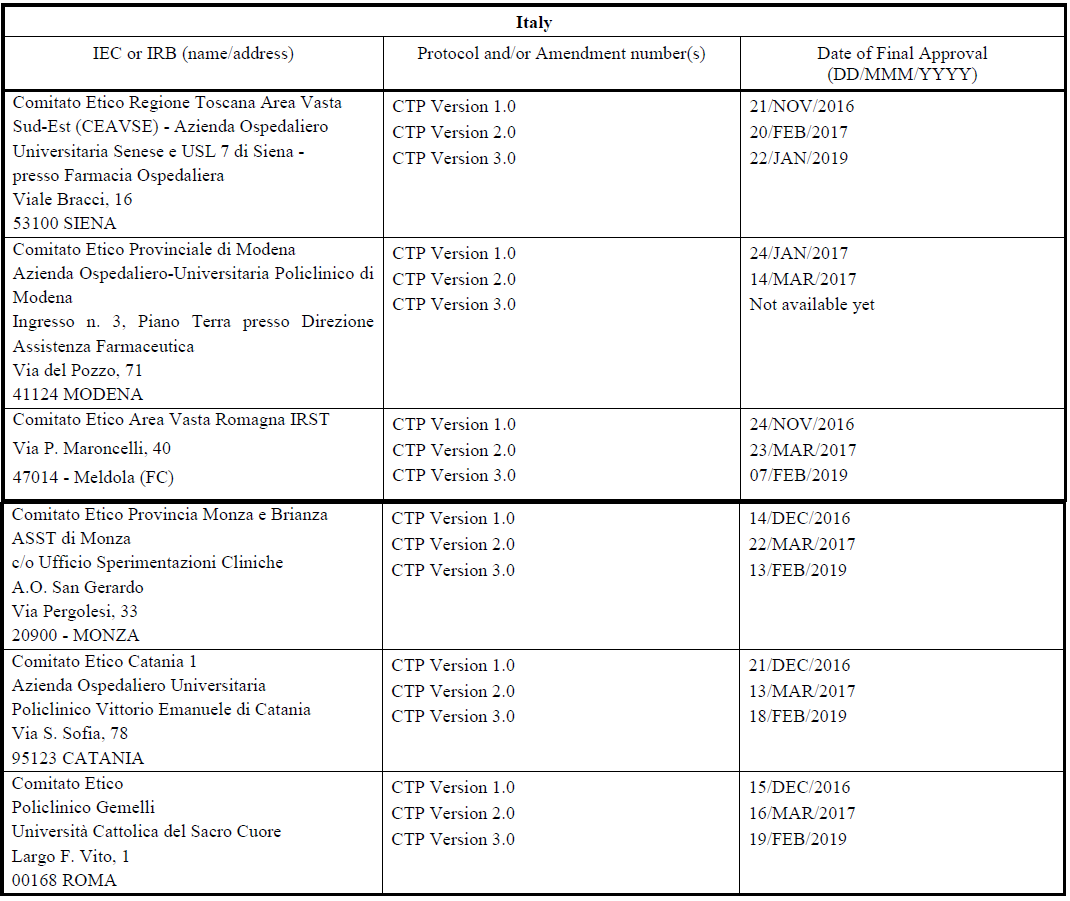
**

**
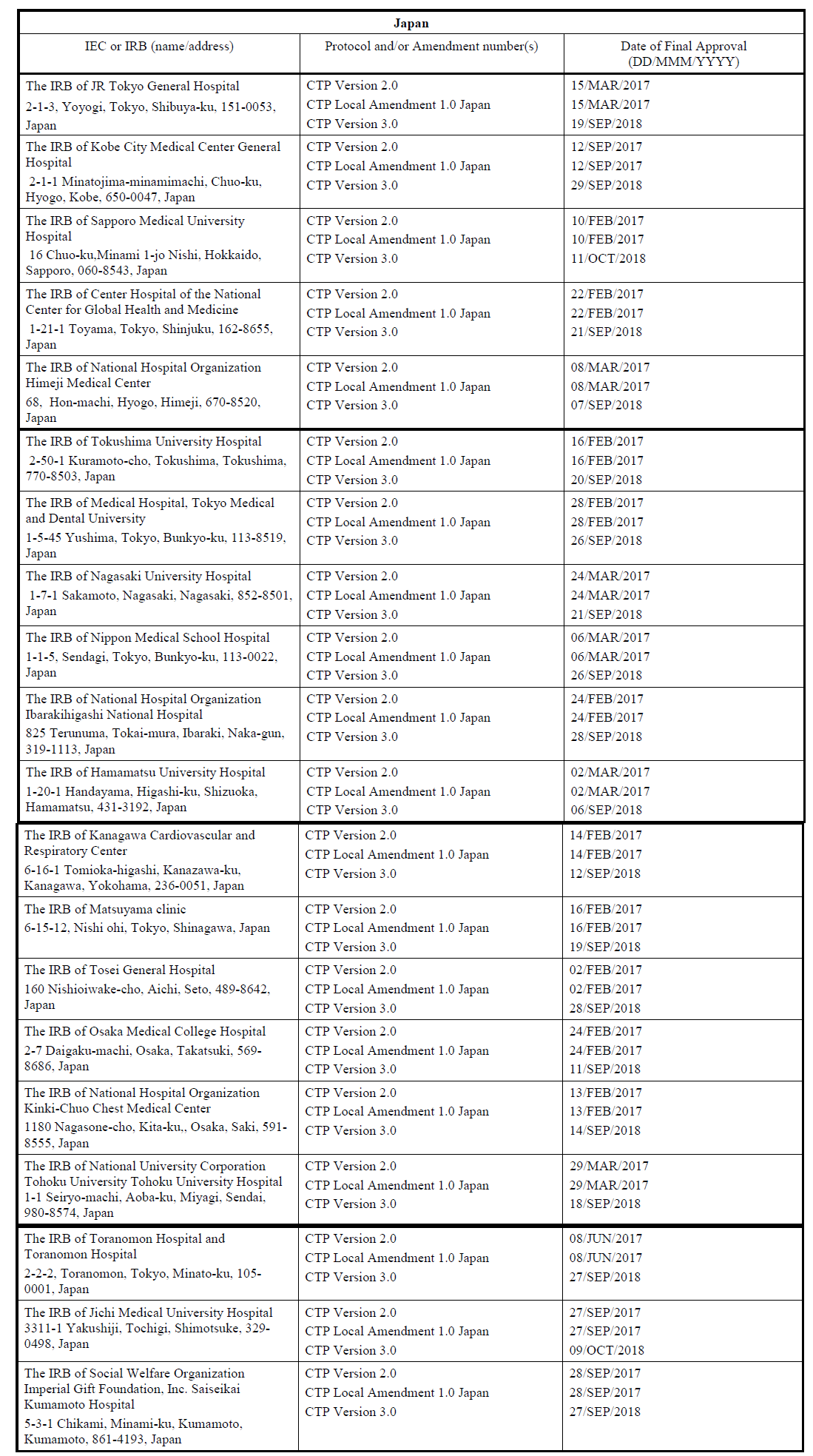
**


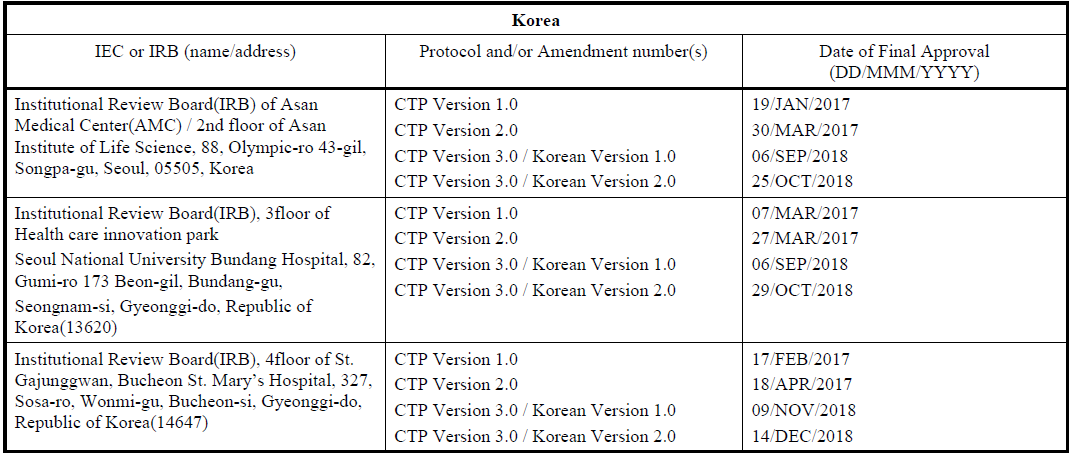


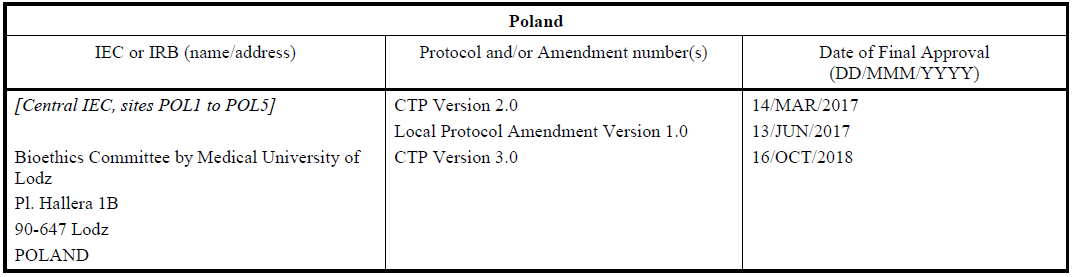


**
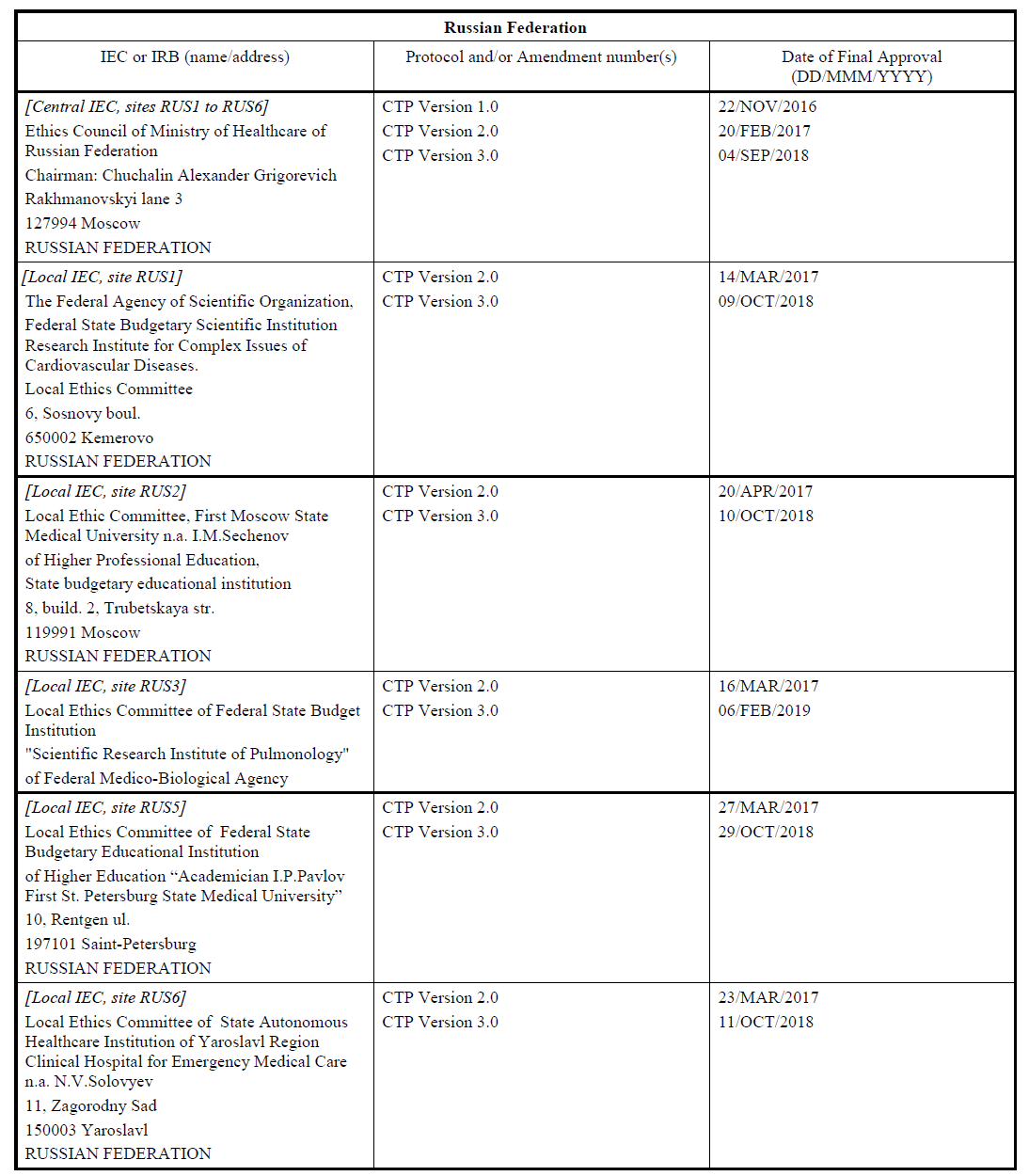
**


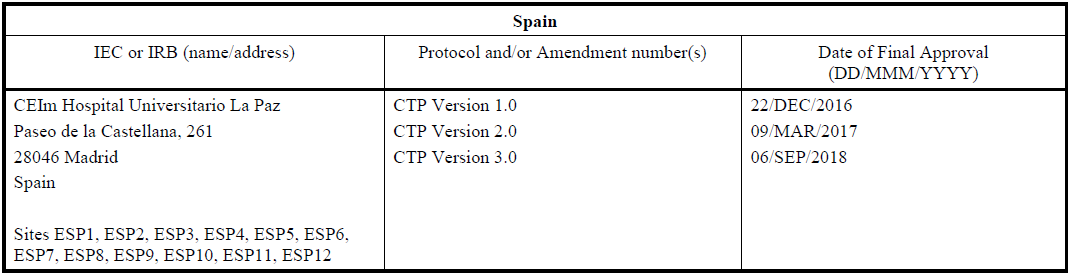


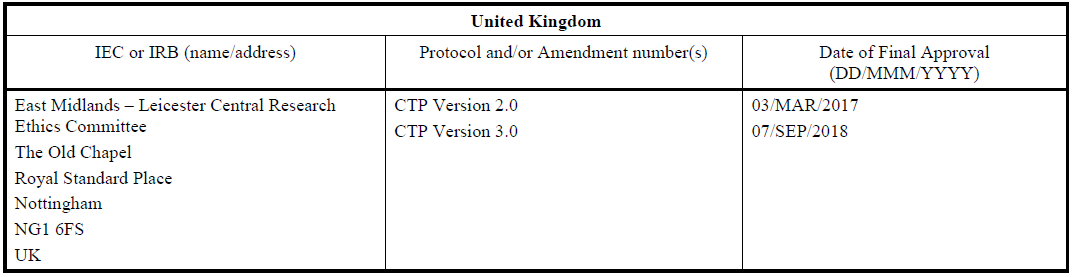


**
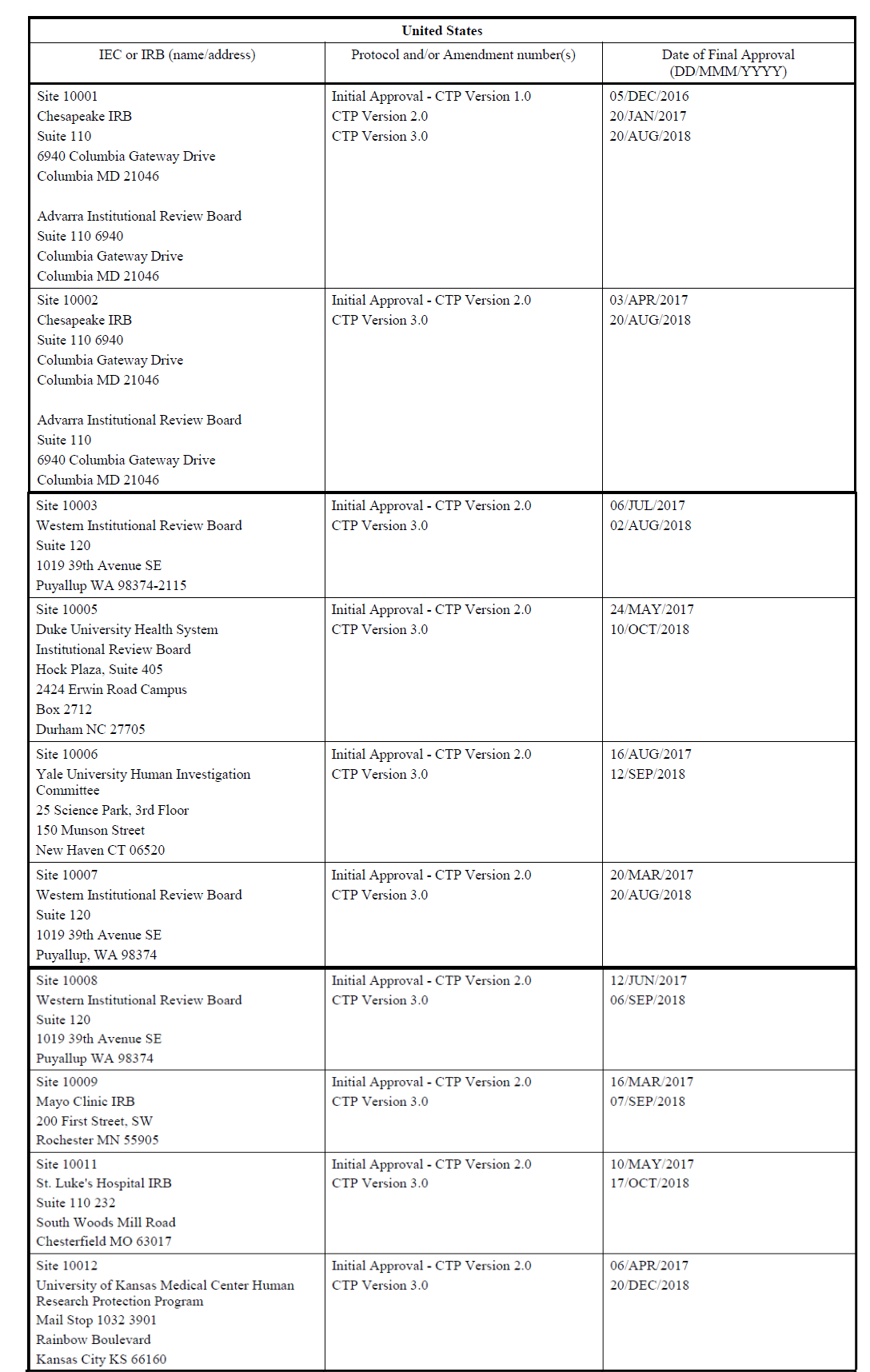

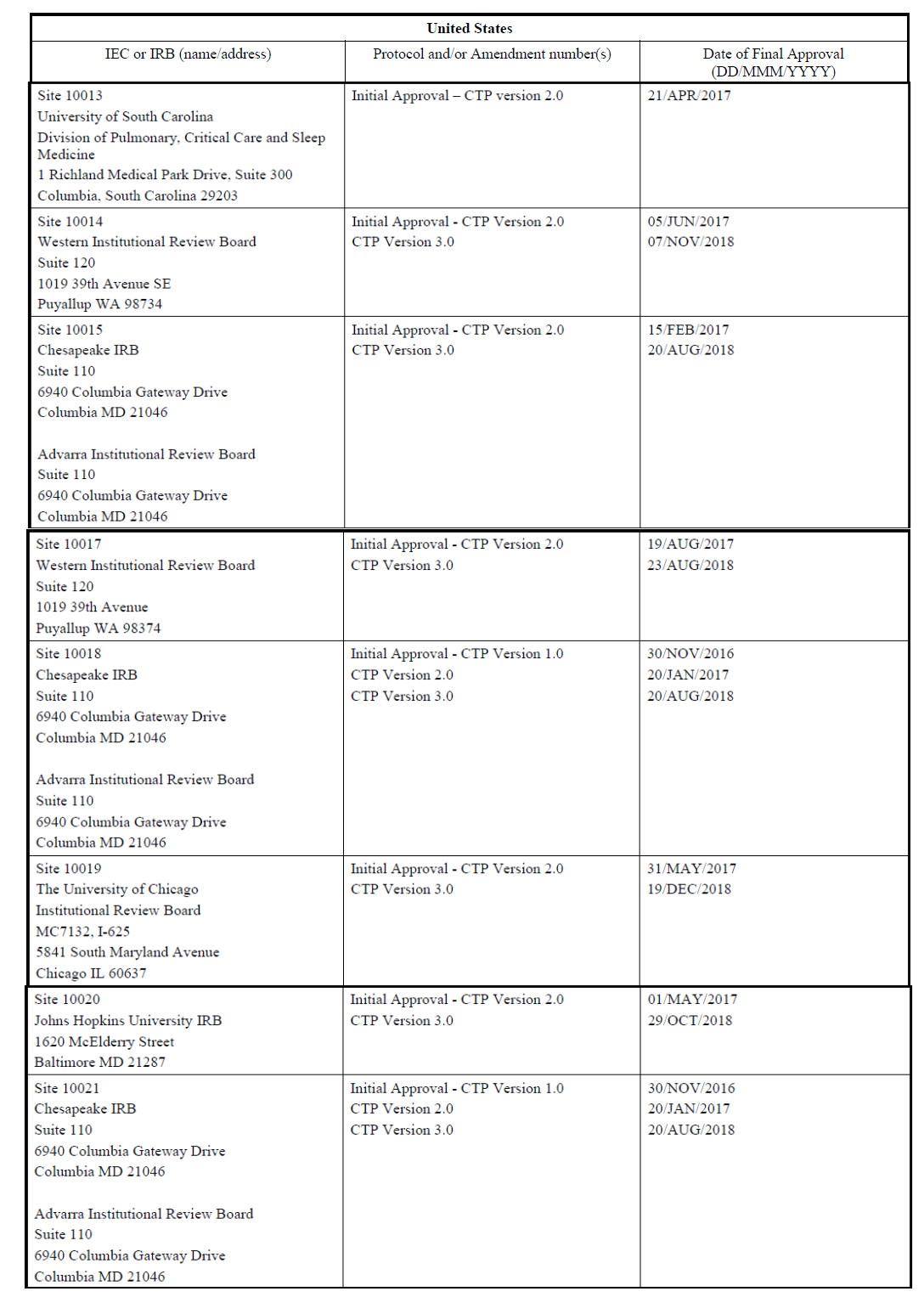
**

**
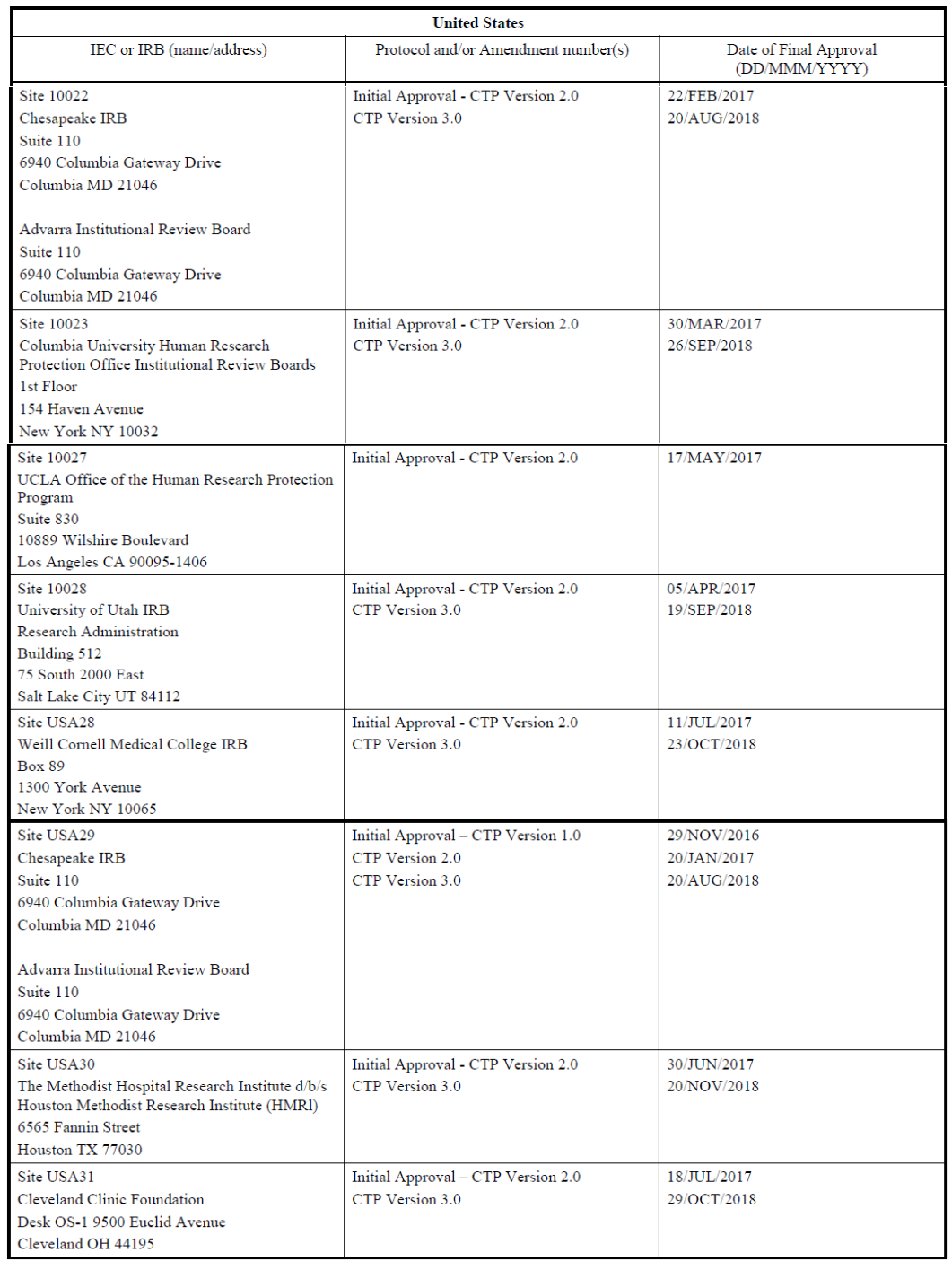
**

**
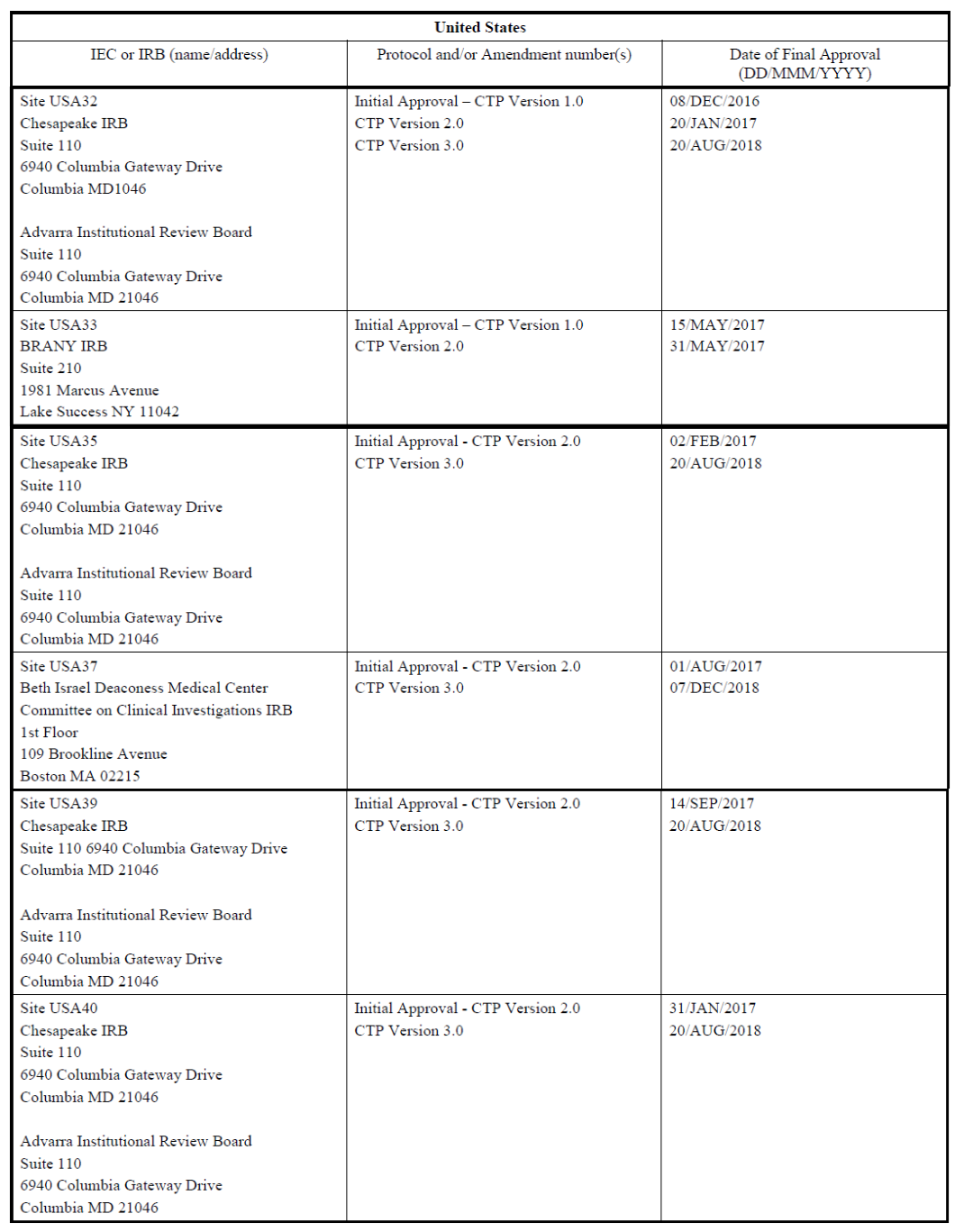
**

**
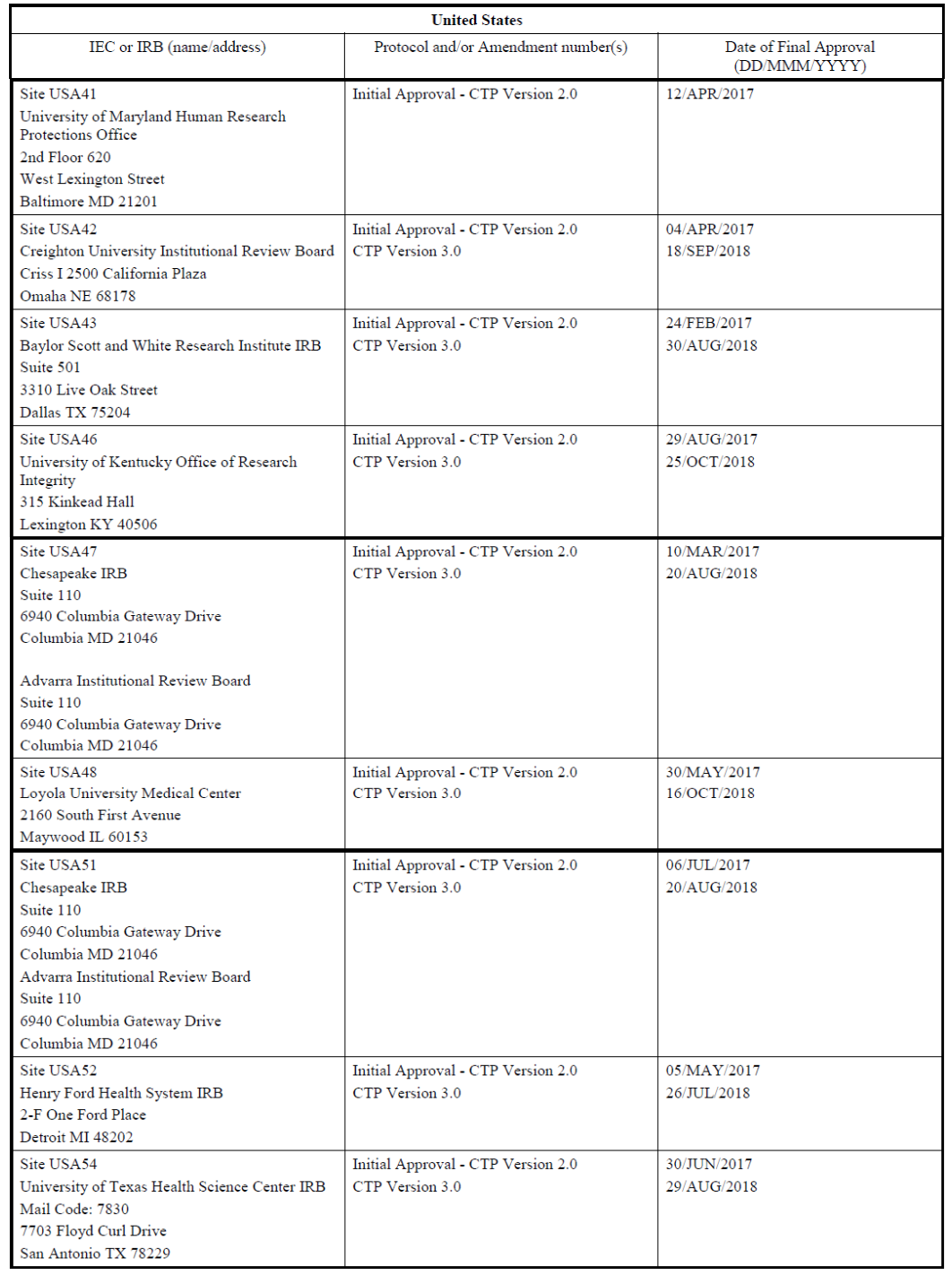
**

**
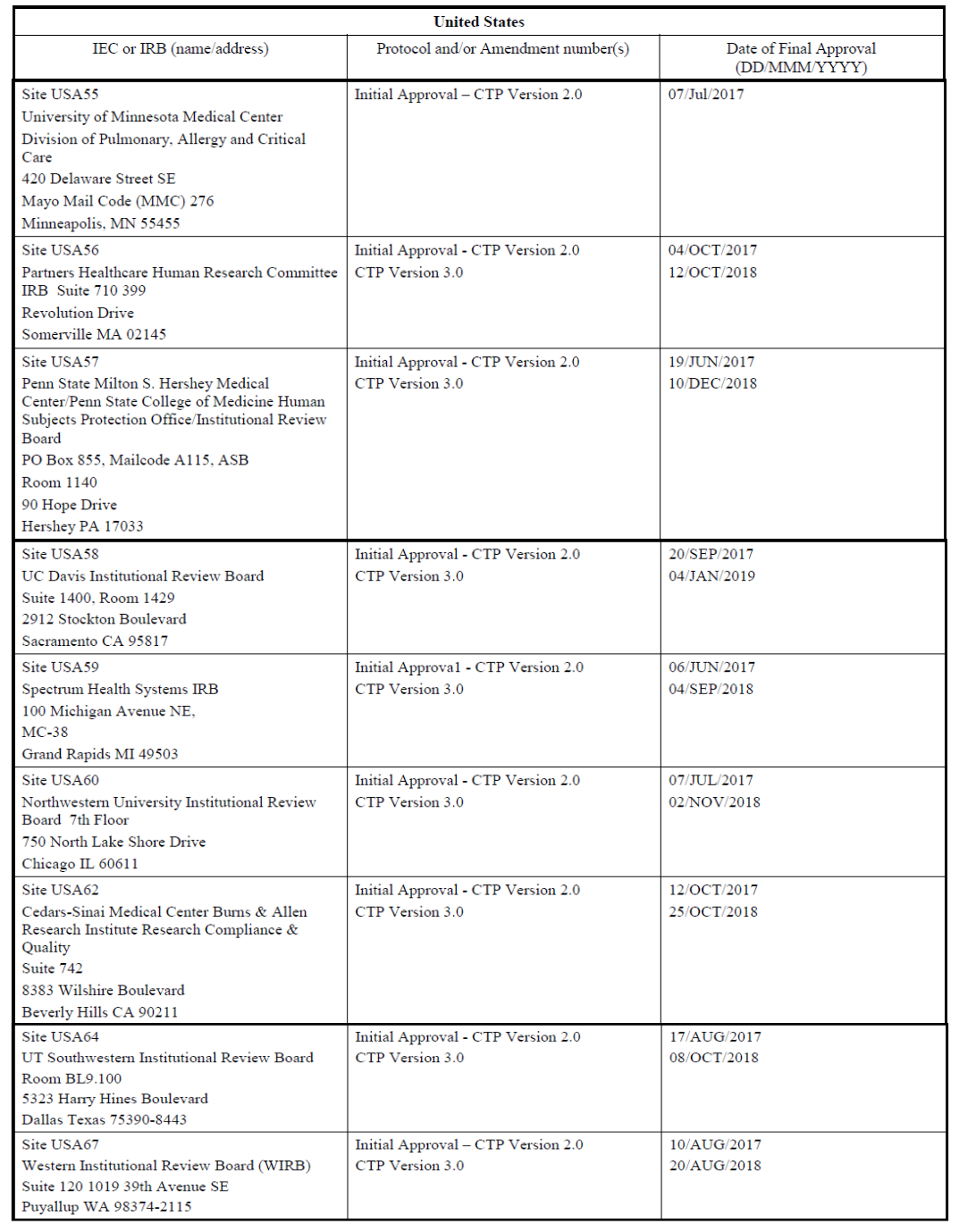
**
