# Supplementary material for: Nintedanib and immunomodulatory therapies in progressive fibrosing interstitial lung diseases
Source: Respir Res. 2021 Mar 16;22:84. doi: 10.1186/s12931-021-01668-1 (PMC7962343; doi:10.1186/s12931-021-01668-1)
Supplement: Supplementary file 2 — Additional file 2: Appendix S2. Analysis of the annual rate of decline in FVC (mL/year). [file 12931_2021_1668_MOESM2_ESM.docx]

**Supplemental Appendix 2:** Analysis of the annual rate of decline in FVC (mL/year)

In the overall population, the annual rate of decline in FVC (mL/year) was analyzed using a random coefficient regression model (with random slopes and intercepts) including treatment, HRCT pattern (UIP-like fibrotic pattern or other fibrotic patterns), baseline FVC (mL), and treatment-by-time and baseline-by-time interactions. Statistical tests were based on least-squares means using a two-sided α = 0.05 (two-sided 95% confidence intervals). The treatment comparison of slopes was assessed through the treatment-by-time interaction coefficient.

Subgroup analyses in the overall population were performed using a random coefficient regression model (with random slopes and intercepts) including baseline FVC (mL), HRCT pattern (UIP-like fibrotic pattern or other fibrotic patterns), and baseline−by−time, treatment-by-subgroup and treatment-by-subgroup-by-time interactions. Nominal *P* values for the treatment-by-subgroup-by-time interaction were obtained from tests of heterogeneity across all expression levels of subgroupings, with no adjustment for multiple testing. The same models were used for analyses in subjects with a UIP-like fibrotic pattern and with other fibrotic patterns on HRCT, except that HRCT pattern was not included as a term. For analyses on a restricted set of these three populations, the same models were used as for the respective complete population. Other than the primary analysis in subjects with a UIP-like fibrotic pattern, all p-values were considered nominal.

The relative treatment effect of nintedanib on slowing ILD progression and the corresponding 95% confidence intervals were derived based on the absolute treatment effects, which were normalized by the adjusted annual rate of decline in FVC in the placebo group. An exploratory meta-analysis was performed for the subgroups and Cochran’s Q-test was used to test for heterogeneity.
