# Supplementary material for: Nintedanib and immunomodulatory therapies in progressive fibrosing interstitial lung diseases
Source: Respir Res. 2021 Mar 16;22:84. doi: 10.1186/s12931-021-01668-1 (PMC7962343; doi:10.1186/s12931-021-01668-1)
Supplement: Supplementary file 3 — Additional file 3: Table S1. Proportions of subjects taking disease-modifying anti-rheumatic drugs (DMARDs) at baseline by WHO standardized drug grouping and preferred name. [file 12931_2021_1668_MOESM3_ESM.docx]

**Supplemental Table 1.** Proportions of subjects taking disease-modifying anti-rheumatic drugs (DMARDs) at baseline by WHO standardized drug grouping and preferred name.

|  | **Overall population**  **(n=663)** | **UIP-like fibrotic pattern on HRCT**  **(n=412)** | **Other fibrotic patterns on HRCT**  **(n=251)** |
| --- | --- | --- | --- |
| Biologic DMARDs | 31 (4.7) | 27 (6.6) | 4 (1.6) |
| Denosumab | 11 (1.7) | 8 (1.9) | 3 (1.2) |
| Abatacept | 6 (0.9) | 5 (1.2) | 1 (0.4) |
| Etanercept | 4 (0.6) | 4 (1.0) | 0 |
| Tocilizumab | 4 (0.6) | 4 (1.0) | 0 |
| Adalimumab | 3 (0.5) | 3 (0.7) | 0 |
| Infliximab | 2 (0.3) | 2 (0.5) | 0 |
| Rituximab* | 1 (0.2) | 1 (0.2) | 0 |
| Ascorbic acid; collagen | 1 (0.2) | 0 | 1 (0.4) |
| Other | 1 (0.2) | 1 (0.2) | 0 |
| Non-biologic DMARDs | 77 (11.6) | 61 (14.8) | 16 (6.4) |
| Hydroxychloroquine | 22 (3.3) | 18 (4.4) | 4 (1.6) |
| Leflunomide | 18 (2.7) | 16 (3.9) | 2 (0.8) |
| Methotrexate | 15 (2.3) | 10 (2.4) | 5 (2.0) |
| Sulfasalazine | 10 (1.5) | 9 (2.2) | 1 (0.4) |
| Hydroxychloroquine sulfate | 8 (1.2) | 6 (1.5) | 2 (0.8) |
| Methotrexate sodium | 4 (0.6) | 4 (1.0) | 0 |
| Mycophenolate mofetil* | 3 (0.5) | 3 (0.7) | 0 |
| Chloroquine phosphate | 2 (0.3) | 1 (0.2) | 1 (0.4) |
| Ciclosporin* | 2 (0.3) | 2 (0.5) | 0 |
| Doxycycline | 2 (0.3) | 2 (0.5) | 0 |
| Penicillamine | 2 (0.3) | 1 (0.2) | 1 (0.4) |
| Bucillamine | 1 (0.2) | 1 (0.2) | 0 |
| Iguratimod | 1 (0.2) | 1 (0.2) | 0 |
| Minocycline hydrochloride | 1 (0.2) | 0 | 1 (0.4) |
| Tacrolimus* | 1 (0.2) | 1 (0.2) | 0 |

Data are n (%) of subjects taking ≥1 such therapy at baseline. A patient could be counted in ≥1 category. HRCT = high-resolution computed tomography; UIP = usual interstitial pneumonia. *Deviation from the trial protocol.
