# Supplementary material for: Nintedanib and immunomodulatory therapies in progressive fibrosing interstitial lung diseases
Source: Respir Res. 2021 Mar 16;22:84. doi: 10.1186/s12931-021-01668-1 (PMC7962343; doi:10.1186/s12931-021-01668-1)
Supplement: Supplementary file 4 — Additional file 4: Table S2. Proportions of subjects taking disease-modifying anti-rheumatic drugs (DMARDs) at baseline by WHO standardized drug grouping and preferred name in subgroups by ILD diagnosis. [file 12931_2021_1668_MOESM4_ESM.docx]

**Supplemental Table 2.** Proportions of subjects taking disease-modifying anti-rheumatic drugs (DMARDs) at baseline by WHO standardized drug grouping and preferred name in subgroups by ILD diagnosis.

|  | **Hypersensitivity pneumonitis**  **(n=173)** | **iNSIP**  **(n=125)** | **Unclassifiable IIP (n=114)** | **Autoimmune ILDs***  **(n=170)** | **Other ILDs^†^**  **(n=81)** |
| --- | --- | --- | --- | --- | --- |
| Biologic DMARDs | 7 (4.0) | 1 (0.8) | 1 (0.9) | 21 (12.4) | 1 (1.2) |
| Denosumab | 5 (2.9) | 1 (0.8) | 1 (0.9) | 3 (1.8) | 1 (1.2) |
| Abatacept | 0 | 0 | 0 | 6 (3.5) | 0 |
| Etanercept | 0 | 0 | 0 | 4 (2.4) | 0 |
| Tocilizumab | 0 | 0 | 0 | 4 (2.4) | 0 |
| Adalimumab | 0 | 0 | 0 | 3 (1.8) | 0 |
| Infliximab | 0 | 0 | 0 | 2 (1.2) | 0 |
| Rituximab^‡^ | 0 | 0 | 0 | 1 (0.6) | 0 |
| Ascorbic acid; collagen | 1 (0.6) | 0 | 0 | 0 | 0 |
| Other | 1 (0.6) | 0 | 0 | 0 | 0 |
| Non-biologic DMARDs | 7 (4.0) | 2 (1.6) | 2 (1.8) | 61 (35.9) | 5 (6.2) |
| Hydroxychloroquine | 0 | 0 | 1 (0.9) | 20 (11.8) | 1 (1.2) |
| Leflunomide | 1 (0.6) | 0 | 1 (0.9) | 15 (8.8) | 1 (1.2) |
| Methotrexate | 1 (0.6) | 1 (0.8) | 0 | 11 (6.5) | 2 (2.5) |
| Sulfasalazine | 0 | 0 | 0 | 10 (5.9) | 0 |
| Hydroxychloroquine sulfate | 1 (0.6) | 1 (0.8) | 0 | 6 (3.5) | 0 |
| Methotrexate sodium | 0 | 0 | 0 | 4 (2.4) | 0 |
| Mycophenolate mofetil^‡^ | 1 (0.6) | 0 | 0 | 2 (1.2) | 0 |
| Chloroquine phosphate | 0 | 0 | 0 | 2 (1.2) | 0 |
| Ciclosporin^‡^ | 1 (0.6) | 0 | 1 (0.9) | 0 | 0 |
| Doxycycline | 1 (0.6) | 0 | 0 | 0 | 1 (1.2) |
| Penicillamine | 0 | 0 | 0 | 2 (1.2) | 0 |
| Bucillamine | 0 | 0 | 0 | 1 (0.6) | 0 |
| Iguratimod | 0 | 0 | 0 | 1 (0.6) | 0 |
| Minocycline hydrochloride | 1 (0.6) | 0 | 0 | 0 | 0 |
| Tacrolimus^‡^ | 1 (0.6) | 0 | 0 | 0 | 0 |

Data are n (%) of subjects taking ≥1 such therapy at baseline. A patient could be counted in ≥1 category. *Included RA-ILD, SSc-ILD, MCTD-ILD, plus autoimmune ILDs in “Other fibrosing ILDs” category of case report form. ^†^Included sarcoidosis, exposure-related ILDs and selected other terms in the “Other fibrosing ILDs” category of the case report form. IIP = idiopathic interstitial pneumonia; ILD = interstitial lung disease; iNSIP = idiopathic non-specific interstitial pneumonia. ^‡^Deviation from the trial protocol.
