# Supplementary material for: Nintedanib and immunomodulatory therapies in progressive fibrosing interstitial lung diseases
Source: Respir Res. 2021 Mar 16;22:84. doi: 10.1186/s12931-021-01668-1 (PMC7962343; doi:10.1186/s12931-021-01668-1)
Supplement: Supplementary file 5 — Additional file 5: Table S3. Baseline characteristics of subjects with a UIP-like fibrotic pattern on HRCT taking high-dose, low-dose, or no glucocorticoids at baseline. [file 12931_2021_1668_MOESM5_ESM.docx]

**Supplemental Table 3.** Baseline characteristics of subjects with a UIP-like fibrotic pattern on HRCT taking high-dose, low-dose, or no glucocorticoids at baseline.

|  | **High-dose glucocorticoids**  **(n=3)** | **Low-dose glucocorticoids**  **(n=217)** | **No glucocorticoids**  **(n=192)** |
| --- | --- | --- | --- |
| Male | 1 (33.3) | 129 (59.4) | 117 (60.9) |
| Age, yr | 60.3 (4.7) | 67.8 (8.7) | 68.4 (8.2) |
| Former or current smoker | 2 (66.7) | 120 (55.3) | 114 (59.4) |
| FVC, mL | 1818 (586) | 2304 (672) | 2450 (807) |
| FVC, % predicted | 57.3 (5.7) | 69.0 (15.0) | 72.6 (16.7) |
| DLco, % predicted | 45.2 (7.0) | 44.0 (12.8) | 49.6 (15.4) |

Data are n (%) or mean (SD). Glucocorticoids with oral, intravenous, intravenous bolus, intravenous drip, or intramuscular route of administration. High-dose glucocorticoids: >20 mg/day prednisone or equivalent. DLco = diffusing capacity of the lung for carbon monoxide, corrected for hemoglobin; FVC = forced vital capacity; HRCT = high-resolution computed tomography; UIP = usual interstitial pneumonia.
