# Supplementary material for: Nintedanib and immunomodulatory therapies in progressive fibrosing interstitial lung diseases
Source: Respir Res. 2021 Mar 16;22:84. doi: 10.1186/s12931-021-01668-1 (PMC7962343; doi:10.1186/s12931-021-01668-1)
Supplement: Supplementary file 6 — Additional file 6: Table S4. Baseline characteristics of subjects with other fibrotic patterns on HRCT taking high-dose, low-dose, or no glucocorticoids at baseline. [file 12931_2021_1668_MOESM6_ESM.docx]

**Supplemental Table 4.** Baseline characteristics of subjects with other fibrotic patterns on HRCT taking high-dose, low-dose, or no glucocorticoids at baseline.

|  | **High-dose glucocorticoids**  **(n=5)** | **Low-dose glucocorticoids**  **(n=136)** | **No glucocorticoids (n=110)** |
| --- | --- | --- | --- |
| Male | 1 (20.0) | 60 (44.1) | 48 (43.6) |
| Age, yr | 62.6 (4.2) | 61.9 (10.9) | 62.3 (10.7) |
| Former or current smoker | 1 (20.0) | 53 (39.0) | 48 (43.6) |
| FVC, mL | 1699 (585) | 2261 (734) | 2304 (699) |
| FVC, % predicted | 51.6 (7.2) | 65.0 (14.4) | 68.7 (15.1) |
| DLco, % predicted | 43.9 (11.0) | 45.1 (12.2) | 45.8 (12.9) |

Data are n (%) or mean (SD). Glucocorticoids with oral, intravenous, intravenous bolus, intravenous drip, or intramuscular route of administration. High-dose glucocorticoids: >20 mg/day prednisone or equivalent. DLco = diffusing capacity of the lung for carbon monoxide, corrected for hemoglobin; FVC = forced vital capacity; HRCT = high-resolution computed tomography; UIP = usual interstitial pneumonia.
