# Supplementary material for: Nintedanib and immunomodulatory therapies in progressive fibrosing interstitial lung diseases
Source: Respir Res. 2021 Mar 16;22:84. doi: 10.1186/s12931-021-01668-1 (PMC7962343; doi:10.1186/s12931-021-01668-1)
Supplement: Supplementary file 7 — Additional file 7: Table S5. Rate of decline in forced vital capacity (FVC) (mL/year) over 52 weeks in subgroups taking or not taking glucocorticoids (high-dose or low-dose) at baseline in subjects with a UIP-like fibrotic pattern on HRCT and subjects with other fibrotic patterns on HRCT. [file 12931_2021_1668_MOESM7_ESM.docx]

**Supplemental Table 5.** Rate of decline in forced vital capacity (FVC) (mL/year) over 52 weeks in subgroups taking or not taking glucocorticoids (high-dose or low-dose) at baseline in subjects with a UIP-like fibrotic pattern on HRCT and subjects with other fibrotic patterns on HRCT. High-dose glucocorticoids: >20 mg/day prednisone or equivalent. HRCT = high-resolution computed tomography; UIP = usual interstitial pneumonia.

|  | **Glucocorticoids (high-dose or low-dose)** | | **No glucocorticoids** | |
| --- | --- | --- | --- | --- |
|  | **Nintedanib** | **Placebo** | **Nintedanib** | **Placebo** |
| **UIP-like fibrotic pattern on HRCT** |  |  |  |  |
| N analyzed | 107 | 113 | 99 | 93 |
| Adjusted mean (SE) rate of decline in FVC (mL/year) over 52 weeks | -81.4 (28.7) | -254.1 (28.4) | -84.6 (30.2) | -164.6 (29.5) |
| Difference vs placebo (95% CI) | 172.7 (93.4, 252.0) | | 80.0 (-2.8, 162.8) | |
| Treatment-by-subgroup-by-time interaction | *P*=0.11 | | | |
| **Other fibrotic patterns on HRCT** |  |  |  |  |
| N analyzed | 70 | 71 | 56 | 54 |
| Adjusted mean (SE) rate of decline in FVC (mL/year) over 52 weeks | -62.2 (29.3) | -145.0 (27.9) | -99.5 (32.3) | -167.0 (32.8) |
| Difference vs placebo (95% CI) | 82.8 (3.2, 162.4) | | 67.5 (-23.3, 158.3) | |
| Treatment-by-subgroup-by-time interaction | *P*=0.80 | | | |
