# Supplementary material for: Nintedanib and immunomodulatory therapies in progressive fibrosing interstitial lung diseases
Source: Respir Res. 2021 Mar 16;22:84. doi: 10.1186/s12931-021-01668-1 (PMC7962343; doi:10.1186/s12931-021-01668-1)

**Supplemental Figure 1.** Rate of decline in forced vital capacity (FVC) (mL/year) over 52 weeks in subgroups taking or not taking glucocorticoids (high-dose or low-dose) at baseline in the overall population (A), in subjects with a UIP-like fibrotic pattern on HRCT (B) and in subjects with other fibrotic patterns on HRCT (C). High-dose glucocorticoids: >20 mg/day prednisone or equivalent. HRCT = high-resolution computed tomography; UIP = usual interstitial pneumonia

**A** Overall population

**
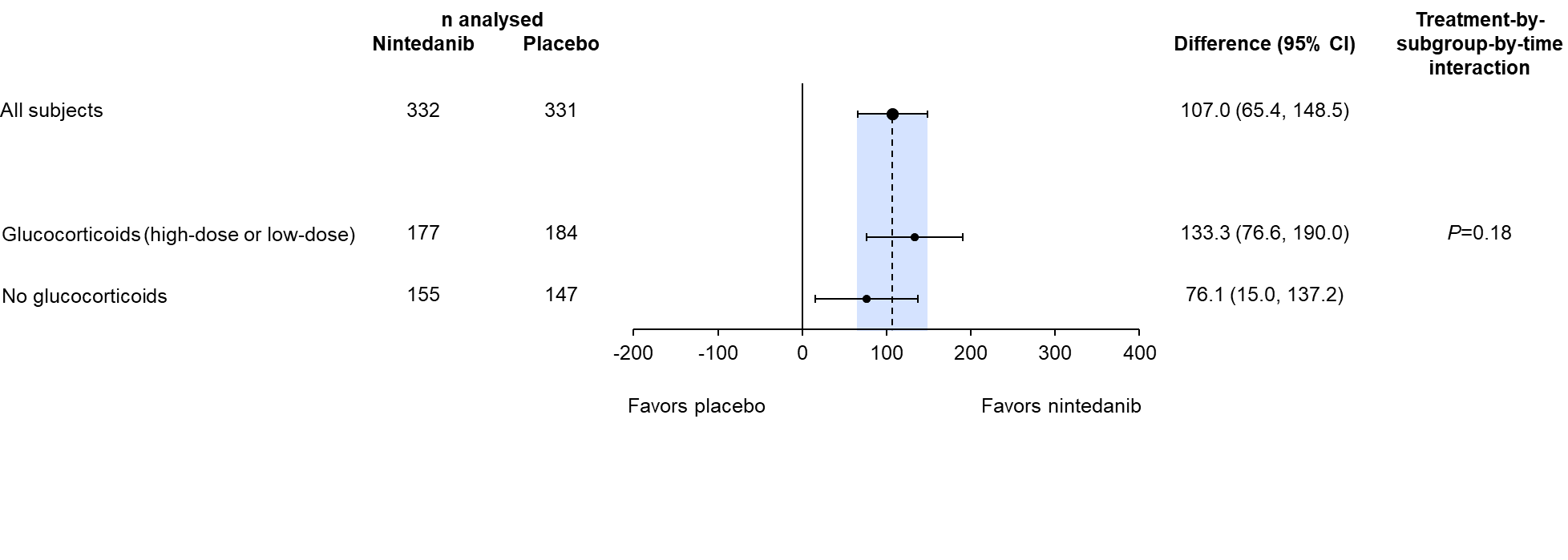
**

**B** Subjects with a UIP-like fibrotic pattern on HRCT


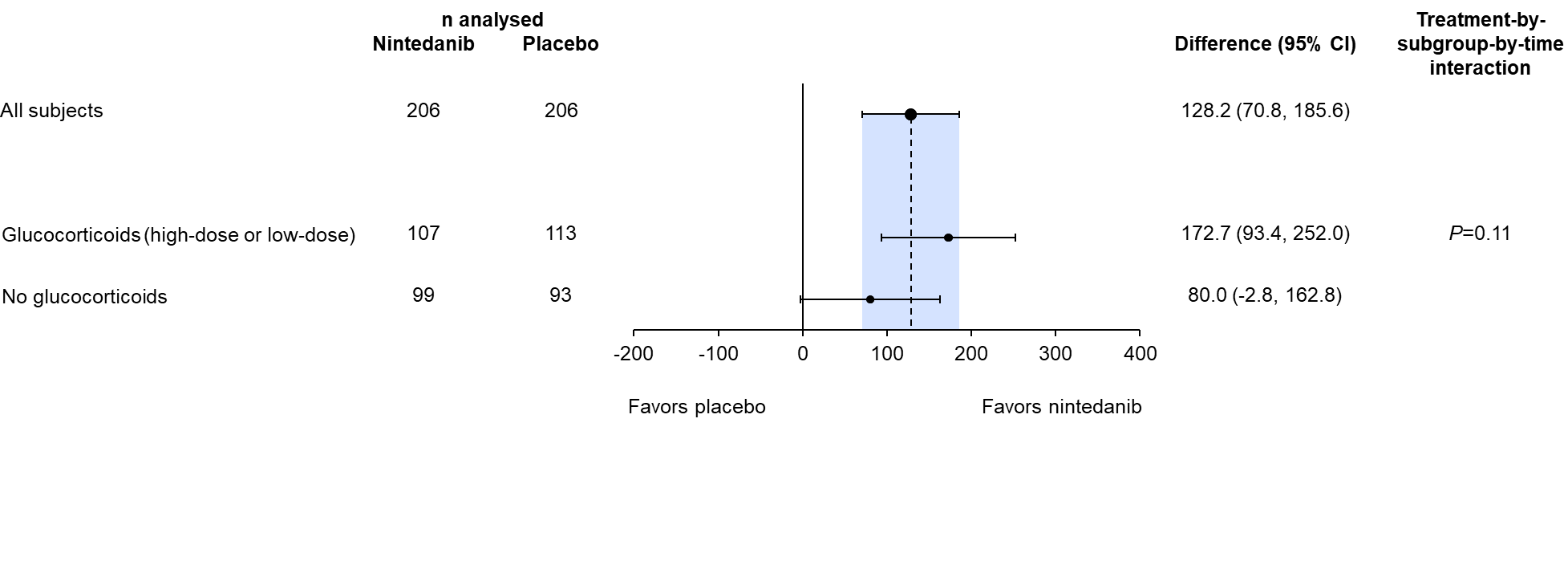


**C** Subjects with other fibrotic patterns on HRCT


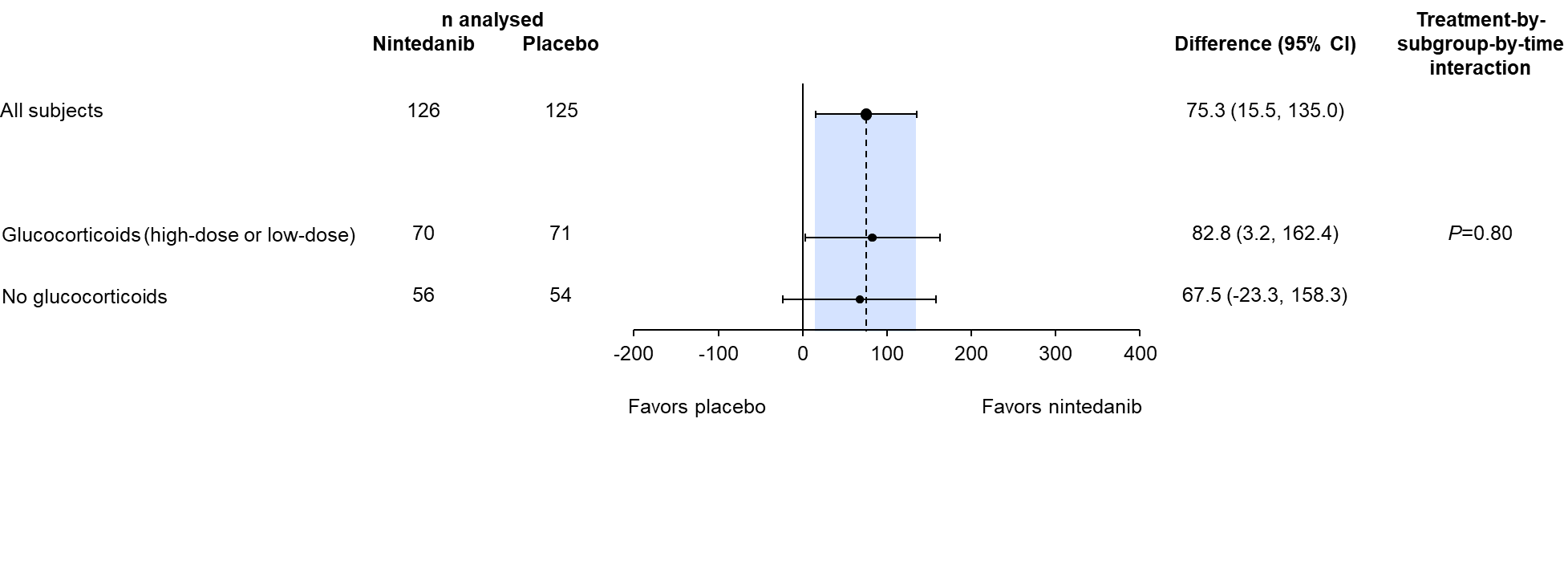

Supplement: Supplementary file 8 — Additional file 8: Figure S1. Rate of decline in forced vital capacity (FVC) (mL/year) over 52 weeks in subgroups taking or not taking glucocorticoids (high-dose or low-dose) at baseline in the overall population (A), in subjects with a UIP-like fibrotic pattern on HRCT (B) and in subjects with other fibrotic patterns on HRCT (C). [file 12931_2021_1668_MOESM8_ESM.docx]
