# Supplementary material for: Nintedanib and immunomodulatory therapies in progressive fibrosing interstitial lung diseases
Source: Respir Res. 2021 Mar 16;22:84. doi: 10.1186/s12931-021-01668-1 (PMC7962343; doi:10.1186/s12931-021-01668-1)
Supplement: Supplementary file 10 — Additional file 10: Table S6. Restricted or prohibited immunomodulatory or antifibrotic therapies taken at baseline, during treatment with trial drug and/or following discontinuation of trial drug over 52 weeks by customized drug grouping or preferred name in subjects with a UIP-like fibrotic pattern on HRCT. [file 12931_2021_1668_MOESM10_ESM.docx]

**Supplemental Table 6.** Restricted or prohibited immunomodulatory or antifibrotic therapies taken at baseline, during treatment with trial drug and/or following discontinuation of trial drug over 52 weeks by customized drug grouping or preferred name in subjects with a UIP-like fibrotic pattern on HRCT.

|  | **Nintedanib**  **(n=206)** | **Placebo**  **(n=206)** |
| --- | --- | --- |
| ≥1 restricted or prohibited therapy | 35 (17.0) | 58 (28.2) |
| Glucocorticoids* | 28 (13.6) | 45 (21.8) |
| Mycophenolate mofetil | 4 (1.9) | 5 (2.4) |
| Azathioprine | 3 (1.5) | 3 (1.5) |
| Tacrolimus | 2 (1.0) | 4 (1.9) |
| Ciclosporin | 0 | 6 (2.9) |
| Cyclophosphamide | 0 | 3 (1.5) |
| Rituximab | 1 (0.5) | 1 (0.5) |
| Nintedanib^*^ | 0 | 2 (1.0) |
| Pirfenidone^*^ | 2 (1.0) | 1 (0.5) |

Data are n (%) of subjects who took ≥1 such therapy at baseline, during treatment with trial drug, and/or following discontinuation of trial drug (up to week 52) for any duration. Glucocorticoids were only counted as restricted therapies if used at high dose (>20 mg/day prednisone or equivalent) and if the route of administration was oral, intravenous, intravenous bolus, intravenous drip, or intramuscular. Other therapies are displayed regardless of dose or route of administration. *Based on customized drug grouping; for other therapies, preferred names are shown. HRCT = high-resolution computed tomography; ILD = interstitial lung disease; UIP = usual interstitial pneumonia.
