# Supplementary material for: Nintedanib and immunomodulatory therapies in progressive fibrosing interstitial lung diseases
Source: Respir Res. 2021 Mar 16;22:84. doi: 10.1186/s12931-021-01668-1 (PMC7962343; doi:10.1186/s12931-021-01668-1)
Supplement: Supplementary file 11 — Additional file 11: Table S7. Restricted or prohibited immunomodulatory or antifibrotic therapies taken at baseline, during treatment with trial drug and/or following discontinuation of trial drug over 52 weeks by customized drug grouping (CDG) category or preferred name in subjects with other fibrotic patterns on HRCT. [file 12931_2021_1668_MOESM11_ESM.docx]

**Supplemental Table 7.** Restricted or prohibited immunomodulatory or antifibrotic therapies taken at baseline, during treatment with trial drug and/or following discontinuation of trial drug over 52 weeks by customized drug grouping (CDG) category or preferred name in subjects with other fibrotic patterns on HRCT.

|  | **Nintedanib (n=126)** | **Placebo**  **(n=125)** |
| --- | --- | --- |
| ≥1 restricted or prohibited therapy | 18 (14.3) | 33 (26.4) |
| Glucocorticoids* | 16 (12.7) | 27 (21.6) |
| Mycophenolate mofetil | 5 (4.0) | 4 (3.2) |
| Azathioprine | 1 (0.8) | 3 (2.4) |
| Tacrolimus | 2 (1.6) | 1 (0.8) |
| Rituximab | 2 (1.6) | 1 (0.8) |
| Ciclosporin | 1 (0.8) | 0 |
| Cyclophosphamide | 0 | 0 |
| Nintedanib^*^ | 0 | 1 (0.8) |
| Pirfenidone^*^ | 0 | 0 |

Data are n (%) of subjects who took ≥1 such therapy at baseline, during treatment with trial drug, and/or following discontinuation of trial drug (up to week 52) for any duration. Glucocorticoids were only counted as restricted therapies if used at high dose (>20 mg/day prednisone or equivalent) and if the route of administration was oral, intravenous, intravenous bolus, intravenous drip, or intramuscular. Other therapies are displayed regardless of dose or route of administration. *Based on CDG category; for other therapies, preferred names are shown. HRCT = high-resolution computed tomography; ILD = interstitial lung disease; MCTD = mixed connective tissue disease; RA = rheumatoid arthritis; SSc = systemic sclerosis.
