# Supplementary material for: Nintedanib and immunomodulatory therapies in progressive fibrosing interstitial lung diseases
Source: Respir Res. 2021 Mar 16;22:84. doi: 10.1186/s12931-021-01668-1 (PMC7962343; doi:10.1186/s12931-021-01668-1)
Supplement: Supplementary file 12 — Additional file 12: Table S8. Restricted or prohibited immunomodulatory or antifibrotic therapies taken at baseline, during treatment with trial drug and/or following discontinuation of trial drug over 52 weeks by customized drug grouping or preferred name in subgroups by ILD diagnosis. [file 12931_2021_1668_MOESM12_ESM.docx]

**Supplemental Table 8.** Restricted or prohibited immunomodulatory or antifibrotic therapies taken at baseline, during treatment with trial drug and/or following discontinuation of trial drug over 52 weeks by customized drug grouping or preferred name in subgroups by ILD diagnosis.

|  | **Hypersensitivity pneumonitis** | | **iNSIP** | | **Unclassifiable IIP** | | **Autoimmune ILDs*** | | **Other ILDs**^†^ | |
| --- | --- | --- | --- | --- | --- | --- | --- | --- | --- | --- |
|  | **Nintedanib (n=84)** | **Placebo (n=89)** | **Nintedanib (n=64)** | **Placebo (n=61)** | **Nintedanib (n=64)** | **Placebo (n=50)** | **Nintedanib (n=82)** | **Placebo (n=88)** | **Nintedanib (n=38)** | **Placebo (n=43)** |
| ≥1 restricted or prohibited therapy | 19 (22.6) | 31 (34.8) | 5 (7.8) | 12 (19.7) | 10 (15.6) | 15 (30.0) | 15 (18.3) | 23 (26.1) | 4 (10.5) | 10 (23.3) |
| Glucocorticoids^‡^ | 15 (17.9) | 23 (25.8) | 4 (6.3) | 11 (18.0) | 9 (14.1) | 11 (22.0) | 12 (14.6) | 18 (20.5) | 4 (10.5) | 9 (20.9) |
| Mycophenolate mofetil | 7 (8.3) | 3 (3.4) | 0 | 0 | 1 (1.6) | 0 | 1 (1.2) | 6 (6.8) | 0 | 0 |
| Azathioprine | 1 (1.2) | 3 (3.4) | 1 (1.6) | 1 (1.6) | 0 | 1 (2.0) | 2 (2.4) | 0 | 0 | 1 (2.3) |
| Tacrolimus | 2 (2.4) | 2 (2.2) | 0 | 0 | 1 (1.6) | 2 (4.0) | 1 (1.2) | 1 (1.1) | 0 | 0 |
| Ciclosporin | 1 (1.2) | 3 (3.4) | 0 | 0 | 0 | 3 (6.0) | 0 | 0 | 0 | 0 |
| Rituximab | 1 (1.2) | 1 (1.1) | 0 | 0 | 0 | 0 | 2 (2.4) | 1 (1.1) | 0 | 0 |
| Cyclophosphamide | 0 | 0 | 0 | 1 (1.6) | 0 | 0 | 0.0 | 2 (2.3) | 0 | 0 |
| Nintedanib^‡^ | 0 | 1 (1.1) | 0 | 1 (1.6) | 0 | 0 | 0 | 1 (1.1) | 0 | 0 |
| Pirfenidone^‡^ | 0 | 0 | 0 | 0 | 1 (1.6) | 1 (2.0) | 1 (1.2) | 0 | 0 | 0 |

Data are n (%) of subjects who took ≥1 such therapy at baseline, during treatment with trial drug, and/or following discontinuation of trial drug (up to week 52). Glucocorticoids were only counted as restricted therapies if used at high dose (>20 mg/day prednisone or equivalent) and if the route of administration was oral, intravenous, intravenous bolus, intravenous drip, or intramuscular. Other therapies are displayed regardless of dose or route of administration. *Included RA-ILD, SSc-ILD, MCTD-ILD, plus autoimmune ILDs in “Other fibrosing ILDs” category of case report form. ^†^Included sarcoidosis, exposure-related ILDs and selected other terms in the “Other fibrosing ILDs” category of the case report form. ^†‡^Based on customized drug grouping; for other therapies, preferred names are shown. IIP = idiopathic interstitial pneumonia; ILD = interstitial lung disease; iNSIP = idiopathic non-specific interstitial pneumonia; MCTD = mixed connective tissue disease; RA = rheumatoid arthritis; SSc = systemic sclerosis.
