# Supplementary material for: Nintedanib and immunomodulatory therapies in progressive fibrosing interstitial lung diseases
Source: Respir Res. 2021 Mar 16;22:84. doi: 10.1186/s12931-021-01668-1 (PMC7962343; doi:10.1186/s12931-021-01668-1)
Supplement: Supplementary file 13 — Additional file 13: Table S9. Proportions of subjects taking high-dose or low-dose glucocorticoids at baseline, during treatment and/or following discontinuation of trial drug over 52 weeks. [file 12931_2021_1668_MOESM13_ESM.docx]

**Supplemental** **Table 9.** Proportions of subjects taking high-dose or low-dose glucocorticoids at baseline, during treatment and/or following discontinuation of trial drug over 52 weeks.

|  | **Overall population** | | **UIP-like fibrotic pattern  on HRCT** | | **Other fibrotic patterns  on HRCT** | |
| --- | --- | --- | --- | --- | --- | --- |
|  | **Nintedanib (n=332)** | **Placebo (n=331)** | **Nintedanib (n=206)** | **Placebo (n=206)** | **Nintedanib (n=126)** | **Placebo (n=125)** |
| High-dose glucocorticoids | 44 (13.3) | 72 (21.8) | 28 (13.6) | 45 (21.8) | 16 (12.7) | 27 (21.6) |
| Low-dose glucocorticoids | 174 (52.4) | 152 (45.9) | 103 (50.0) | 91 (44.2) | 71 (56.3) | 61 (48.8) |
| No glucocorticoids | 114 (34.3) | 107 (32.3) | 75 (36.4) | 70 (34.0) | 39 (31.0) | 37 (29.6) |

Data are n (%) of subjects. High-dose glucocorticoids: >20 mg/day prednisone or equivalent.
