# Supplementary material for: Nintedanib and immunomodulatory therapies in progressive fibrosing interstitial lung diseases
Source: Respir Res. 2021 Mar 16;22:84. doi: 10.1186/s12931-021-01668-1 (PMC7962343; doi:10.1186/s12931-021-01668-1)

**Supplemental Figure 3.** Rate of decline in forced vital capacity (FVC) (mL/year) over 52 weeks in subjects with a UIP-like fibrotic pattern on HRCT (A) or other fibrotic patterns on HRCT (B) in the primary analysis, in an analysis excluding subjects who took ≥1 restricted or prohibited therapy, and in an analysis excluding FVC measurements taken after initiation of restricted or prohibited therapy. HRCT = high-resolution computed tomography; UIP = usual interstitial pneumonia.

**A** Subjects with a UIP-like fibrotic pattern on HRCT


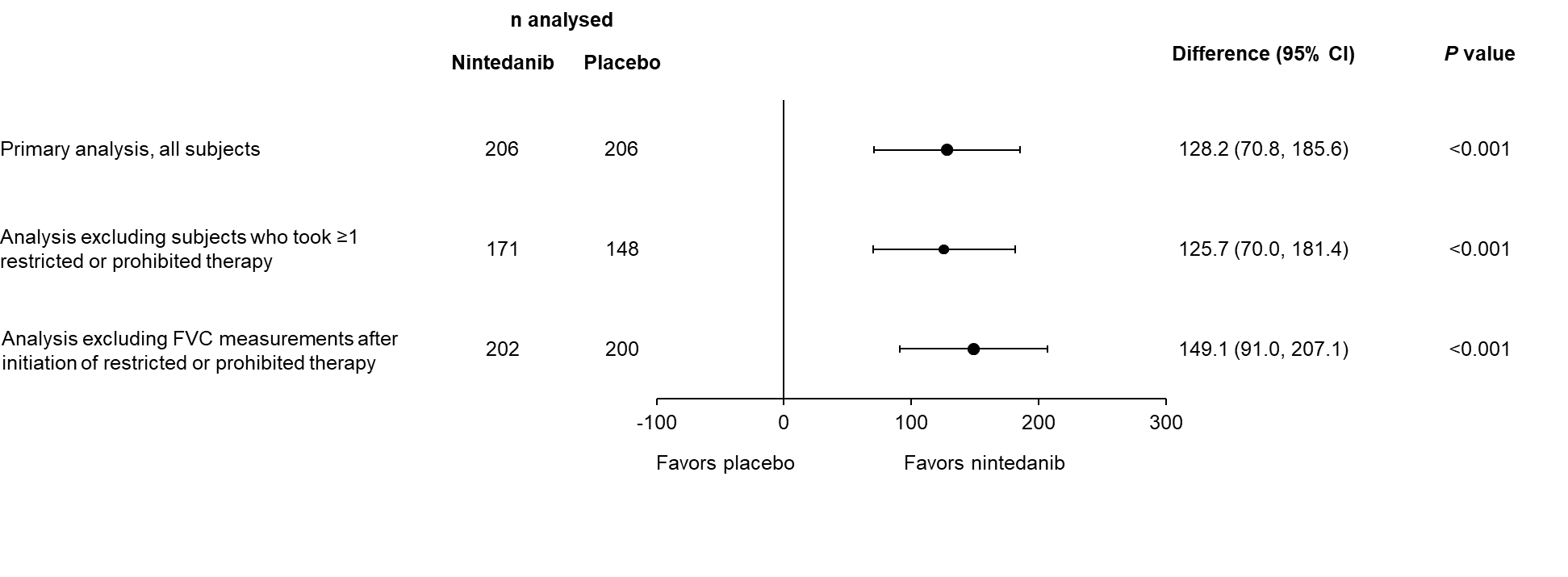


**B** Subjects with other fibrotic patterns on HRCT


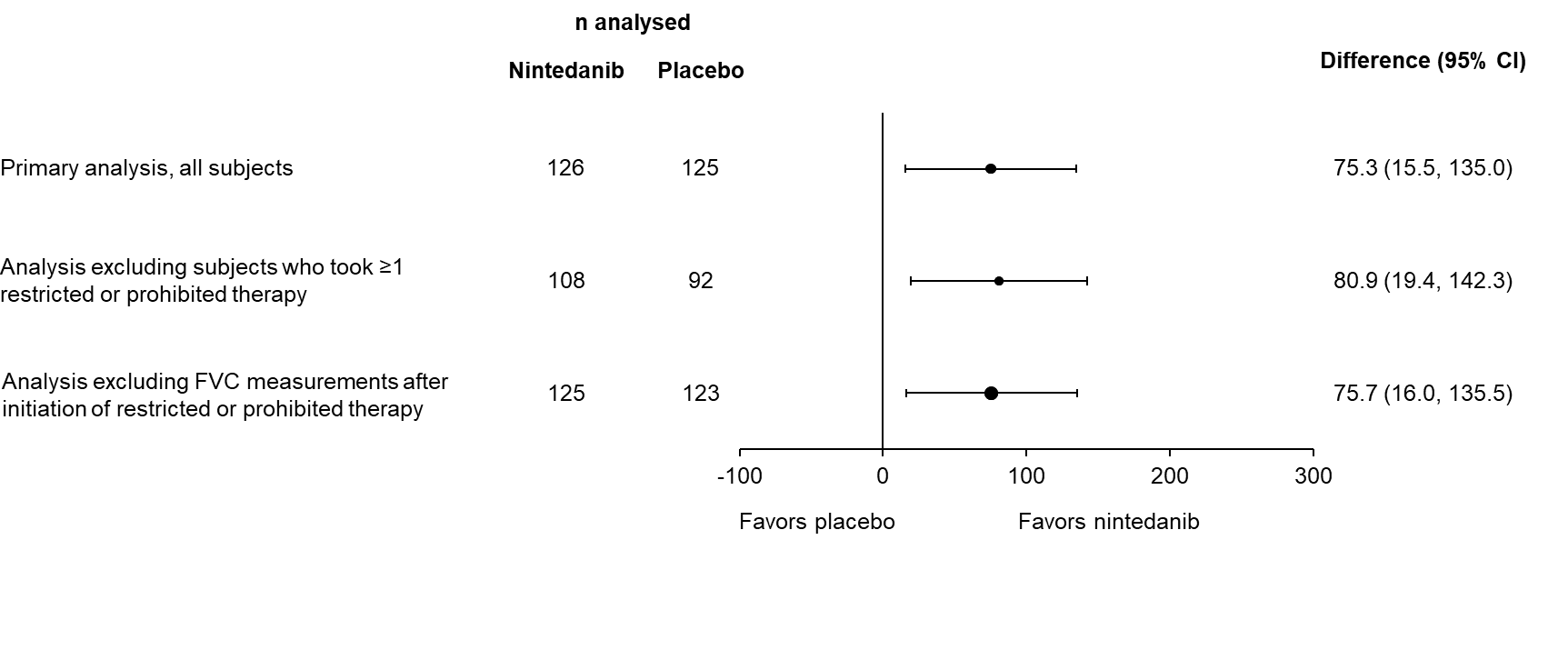

Supplement: Supplementary file 14 — Additional file 14: Figure S3. Rate of decline in forced vital capacity (FVC) (mL/year) over 52 weeks in subjects with a UIP-like fibrotic pattern on HRCT (A) or other fibrotic patterns on HRCT (B) in the primary analysis, in an analysis excluding subjects who took ≥ 1 restricted or prohibited therapy, and in an analysis excluding FVC measurements taken after initiation of restricted or prohibited therapy. [file 12931_2021_1668_MOESM14_ESM.docx]
